# Protonated Glutamate and Aspartate Side Chains Can Recognize Phosphodiester Groups via Strong and Short Hydrogen Bonds in Biomacromolecular Complexes

**DOI:** 10.1002/anie.202501589

**Published:** 2025-05-30

**Authors:** Konstantin Neißner, Elke Duchardt‐Ferner, Christoph Wiedemann, Julian Kraus, Ute A. Hellmich, Jens Wöhnert

**Affiliations:** ^1^ Institute for Molecular Biosciences Goethe‐University Frankfurt/M. Max‐von‐Laue‐Str. 9 60438 Frankfurt Germany; ^2^ Center for Biomolecular Magnetic Resonance (BMRZ) Goethe‐University Frankfurt/M. Max‐von‐Laue‐Str. 9 60438 Frankfurt Germany; ^3^ Institute of Organic Chemistry and Macromolecular Chemistry (IOMC) Friedrich‐Schiller‐University Jena Humboldtstraße 10 07743 Jena Germany; ^4^ Cluster of Excellence ‘Balance of the Microverse’ Friedrich‐Schiller‐University Jena 07743 Jena Germany

**Keywords:** Aspartate, Glutamate, Hydrogen bonding, Phosphodiester group, Protein nucleic acids interactions

## Abstract

Phosphodiester groups occur ubiquitously in nature, e.g. in nucleic acids or in cyclic (di‐)nucleotides important for signal transduction. Proteins often use polar or positively charged amino acids to interact with the negatively charged phosphodiester groups via hydrogen bonds and salt bridges. In contrast, the acidic amino acids aspartate and glutamate are generally not considered as important determinants for phosphodiester group recognition. Instead, they are regarded as detrimental to such interactions due to the assumed charge repulsion between their deprotonated, negatively charged side chain carboxylate groups and the phosphodiester. Accordingly, acidic amino acids are often purposefully introduced into proteins to abrogate nucleic acid interactions in functional studies. Here, we show that in appropriate structural contexts, glutamate side chains are readily protonated even at neutral pH and act as hydrogen bond donors to phosphodiester groups using a c‐di‐GMP binding protein – the GSPII‐B domain of PilF from *Thermus thermophilus* – as an example. Surveying available RNA‐protein and DNA‐protein complex structures in the PDB, we found that hydrogen bonds between apparently protonated carboxylate groups of glutamate and aspartate and phosphodiester groups occur frequently in many different functional protein classes. Thus, the functional role of acidic amino acids in phosphodiester group recognition needs to be re‐evaluated.

## Graphical abstract

The highly negatively charged phosphodiester groups of nucleic acids and cyclic (di‐)nucleotides often interact with amino acids containing polar or positively charged sidechains. Due to charge repulsion with their negatively charged carboxylate groups in their side chains, glutamate and aspartate are considered to be detrimental to such interactions. Here, we show that glutamate residues with pK_a_ values shifted towards neutral pH due to their chemical environment are easily protonated and thus able to form strong hydrogen bonds with phosphodiester groups.

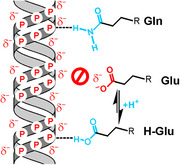

## Introduction

The individual nucleotide building blocks in all DNA and RNA molecules are covalently linked by phosphodiester groups. Phosphodiester groups also occur in cyclic nucleotides and dinucleotides, which serve as important second messengers in cellular signal transduction pathways across all kingdoms of life.

The pK_a_ values for the phosphodiester groups in cyclic mono‐ and dinucleotides as well as in DNA and RNA are reported to be ∼ 1.^[^ ^1^ ^]^ Under physiological pH conditions, these groups are generally deprotonated and negatively charged with the charge delocalized across the two non‐bonding oxygen atoms of the phosphodiester group. Therefore, phosphodiester groups are in principle strong hydrogen bond acceptor groups and simultaneously able to form salt bridges with positively charged functional groups. Particularly strong, charge‐assisted hydrogen bonds can be formed with functional groups that are both positively charged and hydrogen bond donors.

Proteins that bind to phosphodiester groups in DNA, RNA, or cyclic (di‐)nucleotides to regulate gene expression or transmit cellular signals often rely on the polar side chains of amino acids such as serine, threonine, glutamine, asparagine, or tyrosine to act as hydrogen bond donors, or on the positively charged side chains of the basic amino acids arginine and lysine to simultaneously form salt bridges and charge‐assisted hydrogen bonds with their targets.^[^ ^2^, ^3^, ^4^, ^5^, ^6^, ^7^, ^8^ ^]^

In DNA‐protein complexes, such interactions contribute mainly to the binding affinity due to the regular spacing of the phosphodiester groups along the backbone of the DNA double helix,^[^ ^9^ ^]^ while in RNA‐protein complexes, such interactions are often important for the shape‐specific and charge‐distribution‐specific recognition of highly structured RNAs.^[^ ^10^, ^11^ ^]^ Notably, a common approach for testing the functional importance of phosphodiester interactions is to mutate the involved amino acids into aspartate or glutamate. This is based on the reported pK_a_ values for their side chain carboxylate groups of ∼ 4.4 and 3.9 for glutamate and aspartate, respectively,^[^ ^12^, ^13^, ^14^ ^]^ and the resulting almost universally accepted assumption that the introduction of these formally negatively charged amino acid side chains will result in charge repulsion and thereby lower the binding affinity or prevent binding altogether.

Here, we use the example of a cyclic dinucleotide binding protein to show that, contrary to common expectations, the carboxylate groups in the side chains of glutamate and aspartate residues can engage in productive hydrogen bonding interactions with phosphodiester groups. The analysis of previously reported structures of nucleic acid/protein complexes shows that such interactions are widespread, thereby presenting a hitherto underappreciated phosphodiester group recognition mode in biomacromolecular complexes.

**Figure 1:**
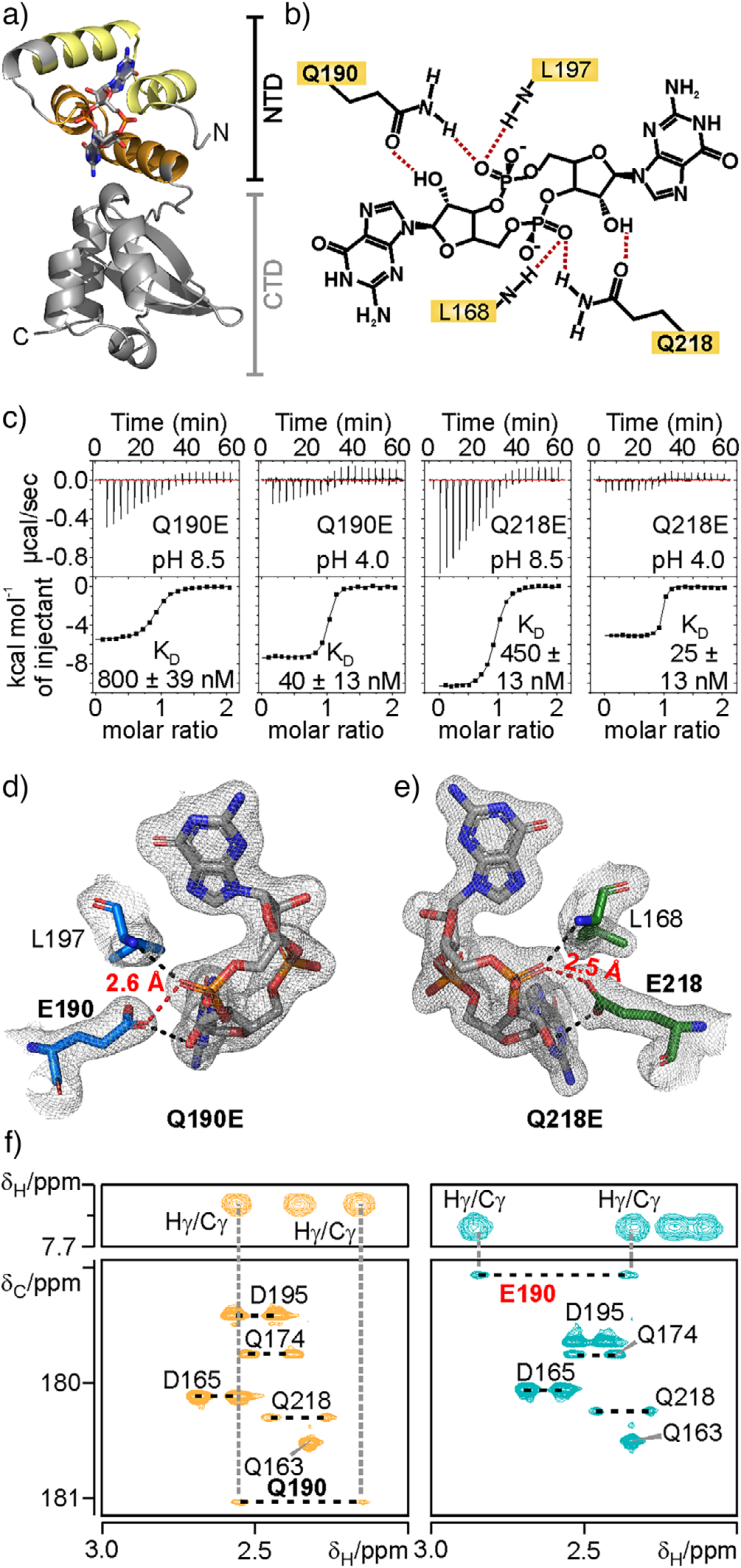
Phosphodiester group recognition in the GSPII‐B mutants Q190E and Q218E is pH‐dependent and relies on a protonated glutamate side chain. a) X‐ray structure of WT GSPII‐B (PilF_159‐302_) in complex with c‐di‐GMP (PDB entry 8pdk).^[^ ^22^ ^]^ The protein is shown in ribbon representation and c‐di‐GMP is shown as sticks with carbon atoms in gray, nitrogen atoms in blue, phosphorous in orange and oxygen in red. The brackets highlight the location of the N‐terminal (NTD) and the C‐terminal (CTD) subdomain of the protein. The first copy of the c‐di‐GMP binding sequence motif is colored yellow, the second orange. b) Schematic representation of the intermolecular hydrogen bonds (dashed red lines) between the GSPII‐B domain and the phosphodiester groups of c‐di‐GMP in the WT protein. Important amino acid residues are labeled.^[^ ^22^ ^]^ c) c‐di‐GMP binding affinities of the Q190E (left) and Q218E (right) mutants at pH values of 8.5 and 4.0 according to ITC measurements. d, e) Intermolecular interactions between the phosphodiester group of c‐di‐GMP and E190 in the mutated binding pocket of the Q190E single‐site mutant (d) and E218 in the mutated binding pocket of the Q218E protein variant (e) according to the X‐ray structures of the two complexes. The electron density at the 1.0 σ level is indicated as a gray mesh. Hydrogen bonds are indicated as black and red dashed lines. f) 2D‐Hγ(Cγ)Cδ NMR spectra of the PilF_159‐221_ WT/c‐di‐GMP complex (left) and the PilF_159‐221_ Q190E/c‐di‐GMP complex (right). The corresponding slices from a 3D‐H(CCO)NH spectrum for the Q190 and E190 side chains, respectively, for both variants in complex with c‐di‐GMP are shown on top. Black dashed lines indicate resonance positions belonging to the Hγ protons from the same methylene group. Gray dashed lines indicate the assignment of the Hγ/Cδ correlation signals via the Hγ/Cγ signals in the slices from the 3D‐H(CCO)NH spectra.

The common bacterial second messenger c‐di‐GMP regulates a wide range of processes, including biofilm formation,^[^ ^15^, ^16^ ^]^ motility,^[^ ^17^ ^]^ and virulence.^[^ ^18^, ^19^ ^]^ One target protein for c‐di‐GMP is PilF, a hexameric ATPase from the thermophilic bacterium *Thermus thermophilus* important for pilus formation, cell motility, and DNA uptake from the environment.^[^ ^20^ ^]^ The N‐terminus of PilF contains three homologous so‐called general secretory pathway II (GSPII) domains, GSPII‐A, ‐B, and ‐C.^[^ ^21^ ^]^ Our recent structures of GSPII‐B (PilF_159‐302_)^[^ ^22^ ^]^ showed that the two nucleotide building blocks of c‐di‐GMP are recognized by the protein in a very similar manner through a duplicated amino acid sequence motif. Both copies of the motif fold into two tightly packed helices (Figure 1a). Together, the two motifs form a four‐helix bundle (Figure 1a) that binds c‐di‐GMP with very high affinity (K_D_ ∼ 6 nM). Other GSPII domain containing proteins bind c‐di‐GMP in the same structurally conserved manner.^[^ ^23^ ^]^

In the GSPII‐B complex with c‐di‐GMP, both c‐di‐GMP phosphodiester groups accept a hydrogen bond from a backbone amide group of a conserved leucine (L168 from the first motif and L197 from the second motif) and from a side chain amino group of a conserved glutamine (Q190 from the first motif and Q218 from the second motif) (Figure 1b). The side chain oxygen atoms of these two glutamines simultaneously serve as hydrogen bond acceptors in hydrogen bonds with the 2′‐OH groups of the adjacent ribose moieties (Figure 1b). Importantly, the intermolecular hydrogen bonds between the negatively charged non‐bridging oxygen atoms of the phosphodiester groups from c‐di‐GMP and polar backbone and side chain groups of GSPII‐B are similar to those commonly observed in protein‐nucleic acid complexes. The high affinity for the ligand, the nature of the bivalent binding motif, and the role of the two conserved glutamine residues make GSPII‐B a well suited model system to investigate the role of sidechain interactions in phosphodiester bond recognition.

## Results and Discussion

We previously individually or simultaneously mutated Q190 and Q218 to glutamate residues (Q190E and Q218E, Figure 1b) in order to purposefully disrupt c‐di‐GMP binding to the GSPII‐B domain by creating electrostatic repulsion between the ligands phosphodiester groups and the carboxylate groups of the glutamate side chains. In vivo, these mutations resulted in reduced bacterial twitching motility and cell adhesion defects.^[^ ^22^ ^]^ In vitro at pH 7.5, which corresponds to the intracellular pH of *T. thermophilus*, c‐di‐GMP did not bind to the double mutant, and showed significantly reduced affinity for the two Q190E and Q218E single‐site mutants as expected.^[^ ^22^ ^]^

However, we were intrigued by the relative orientation of the Q190 and Q218 side chains and the phosphodiester groups to each other in the WT complex (Figure 1b). It suggested that either the protonation of the newly introduced carboxylate group in the Q/E single‐site mutants or the protonation of the phosphodiester group could lead to an energetically favorable hydrogen bonding interaction and a pH‐sensitive protein‐ligand interaction instead of the commonly expected electrostatic repulsive interaction between these two functional groups.

To explore this possibility, we investigated the binding affinity of the two single‐site Q/E mutants (Q190E and Q218E, respectively) over a wider pH range (Figure 1c). While the c‐di‐GMP binding affinity of the WT protein is similar at pH 4.0 and 8.5 according to ITC titration experiments (Figure S1), both mutants display a drastic increase in ligand affinity at lower pH (Figure 1c). The affinity of the Q190E mutant for c‐di‐GMP increased ∼ 20fold with the K_D_ changing from ∼ 800 nM at pH 8.5 to ∼ 40 nM at pH 4.5. Likewise, the K_D_ of the Q218E mutant changed from ∼ 450 nM at pH 8.5 to ∼ 25 nM at pH 4.5. This strongly supports the notion that a protonation event, either within c‐di‐GMP or in the GSPII‐B Q/E single‐site variants, can modulate the ligand‐protein interaction, thereby stabilizing the complex at acidic pH values.

To gain insights into the structural basis for the pH‐dependent ligand recognition, we crystallized both the Q190E and the Q218E single‐site mutant of the PilF GSPII‐B domain in complex with c‐di‐GMP using very similar crystallization conditions at pH 7.0 (Figure S2). Importantly, both protein‐ligand complexes yielded well diffracting crystals and structures were solved at a resolution of 1.9 Å (Q190E, PDB entry 9GL5) and 1.6 Å (Q218E, PDB entry 9GLG), respectively (Table S1). There are no differences in the global structure when comparing the Q190E and Q218E mutants complexed with c‐di‐GMP with each other (global backbone heavy atom RMSD of 0.14 Å) or with the WT (0.15 Å and 0.16 Å, respectively) (Figure S2a, Table S2).

Due to the high‐quality electron density maps for both mutants, their ligand recognition mode could be discerned in detail (Figures 1d,e, S2b,c). In both the Q190E and the Q218E single point mutants, one phosphodiester‐binding pocket can be found in the mutant configuration (Figure 1d,e) and the other one in the WT configuration due to the duplication of the c‐di‐GMP phosphodiester binding motifs (Figure S2b‐e). The comparison of the “WT” binding pockets of the single mutants which is composed of Q218/L168 in the Q190E mutant and Q190/L197 in the Q218E mutant showed that they are structurally highly similar to the equivalent binding pockets in the WT protein, including the orientation of the respective sidechains (Figure S3).

**Figure 2:**
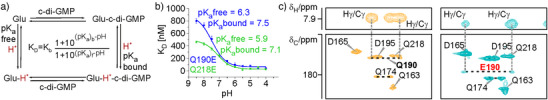
pK_a_ values of the glutamate side chain carboxylate groups in the Q190E and Q218E single mutants in complex with c‐di‐GMP. a) Thermodynamic cycle describing the coupling of c‐di‐GMP binding and protonation of side chain glutamates in the two mutants. The equation describing the experimentally observed KD values as a function of the pK_a_ of the free protein (pK_a_f) and the pK_a_ of the c‐di‐GMP bound protein (pK_a_b) is shown in the center. b) pH dependence of the K_D_ for c‐di‐GMP for the Q190E mutant (blue) and the Q218E mutant (green). c) 2D‐Hγ(Cγ)Cδ NMR spectra of free PilF_159‐221_ WT (left) and free PilF_159‐221_ Q190E (right) with the corresponding slices from the respective 3D‐H(CCO)NH spectra shown on top. Black dashed lines indicate resonance positions belonging to the Hγ protons from the same methylene group. Gray dashed lines indicate the assignment of the Hγ/Cδ correlation signals via the Hγ/Cγ signals in the slices from the 3D‐H(CCO)NH spectra.

Importantly, the carboxylate groups of the newly introduced glutamate sidechains in either of the two Q/E mutants were found to still point directly towards a non‐bridging oxygen atom of the phosphodiester groups (Figure 1d, e), as seen previously for the carboxamide groups of the native glutamine side chains (Figures 1, S2d, e). The shortest distances between the oxygen atoms of the glutamate carboxylate groups and the phosphodiester groups were found to be 2.6 Å (Q190E) and 2.5 Å (Q218E), respectively, which is well below the sum of the van der Waals radii of the two oxygen atoms. For negatively charged carboxylate and phosphodiester groups, this would represent an energetically prohibitive situation due to a strong repulsion of like charges. However, protonation of one of the two groups can alleviate this energetically unfavorable situation by neutralizing one of the charges. In addition, the protonated group could also serve as a hydrogen bond donor while the unprotonated group would be the hydrogen bond acceptor.

To observe such a potential protonated state directly by X‐ray crystallography, a resolution of <1.0 Å would be required.^[^ ^24^ ^]^ Unfortunately, we were not able to optimize our crystallization conditions to this extent. However, NMR‐spectroscopy is a powerful tool to analyze the protonation state of functional groups.^[^ ^25^, ^26^ ^]^ In the case at hand, the ^31^P shifts for the phosphodiester groups of c‐di‐GMP and the ^13^C shifts of the Cδ carbon atoms of the glutamate carboxylate groups are sensitive reporters of their respective protonation states. We first tested the effect of pH on the chemical shift of the ^31^P signals of c‐di‐GMP. Free c‐di‐GMP gives rise to a single ^31^P NMR signal due to its inherent symmetry. Lowering the pH leads to an upfield shift of this signal as an indicator of the (at least partial) protonation of the phosphodiester groups (Figure S4a). In contrast, comparing the ^31^P chemical shifts for c‐di‐GMP bound to the WT protein and the two E/Q mutants reveals a downfield shift for the ^31^P signal of the phosphodiester group close to the glutamate side chain in the mutants compared to those of the phosphodiester groups hydrogen‐bonded to the glutamine side chains of the WT (Figure S4b). This suggests that the respective c‐di‐GMP phosphodiester group is not protonated when bound to the mutant proteins and might instead serve as a hydrogen bond acceptor group.

Next, we aimed to investigate the protonation state of the newly introduced glutamate side chains in the phosphodiester‐binding pocket of GSPII‐B using the Q190E mutant as the model for both Q‐to‐E mutants. To enhance spectral quality and resolution, we used a C‐terminally truncated protein construct containing only the α‐helical N‐terminal subdomain (64 residues, PilF_159‐221_, Figure. 1a). Importantly, the fingerprint ^1^H, ^15^N‐HSQC‐spectra of the isolated subdomain and the full‐length domain are very similar in both the apo‐ and the ligand‐bound states (Figure S5a,b), the c‐di‐GMP‐induced chemical shift changes are similar for the isolated subdomain and the full‐length domain (Figure S5c,d) and the subdomain binds c‐di‐GMP with an affinity comparable to the K_D_ of the full‐length domain,^[^ ^22^ ^]^ showing that the minimal construct faithfully replicates the c‐di‐GMP binding mode of the full‐length GSPII‐B domain.

To elucidate the protonation state of E190 in the Q190E variant bound to c‐di‐GMP, we established the complete backbone (Figure S6a,b) and the relevant side chain NMR signal assignments for both the PilF_159‐221_ WT and the PilF_159‐221_ q190e construct. 3D‐H(CCO)NH and 2D‐Hγ(Cγ)Cδ NMR spectra^[^ ^27^ ^]^ for the WT and the Q190E protein in complex with c‐di‐GMP recorded at pH 5.8 were analyzed and compared to unambiguously identify the resonances belonging to the E190 side chain in the mutant (Figure 1f). By correlating the chemical shifts of the Hγ protons with those of the Cδ carbon atom from the carboxylate group of the same glutamate side chain, the 2D‐Hγ(Cγ)Cδ NMR spectrum allows to interrogate the protonation state of the glutamate side chains (Figures 1f and S7). While unprotonated, negatively charged glutamate carboxylate groups have Cδ chemical shifts of 182–186 ppm, the upfield Cδ chemical shift of ∼ 179 ppm for the E190 side chain (Figure 1f) is indicative for the presence of a neutral protonated carboxylate group.^[^ ^26^ ^]^ It should be noted that the HγCδ correlation signals for E190 are significantly broader and of lower intensity compared to the corresponding correlations for all other glutamate residues in the protein. This is very likely due to an exchange with the residual population of unprotonated E190 which apparently occurs in the intermediate exchange regime on the NMR time scale.

In conjunction with the short distance and the relative orientation between the carboxylate and phosphodiester oxygen atoms observed in the X‐ray structure the upfield chemical shift observed for Cδ of E190 suggests that in the Q190E mutant complex with c‐di‐GMP, the E190 side chain carboxylate group is protonated and forms a strong hydrogen bonding interaction with the phosphodiester group where the protonated carboxylate group of E190 is the hydrogen bond donor and the non‐bonding oxygen of the phosphodiester group is the hydrogen bond acceptor.

The relevance of the unexpected occurrence of a protonated glutamate side chain as an interaction partner for a phosphodiester group at physiological pH is underscored by the fact that our NMR experiments were conducted at pH 5.8, while the crystal structures of the two Q/E mutants bound to c‐di‐GMP were obtained at pH 7.0. To quantitatively characterize this protonation event, we determined the corresponding pK_a_ values for the unbound, and the c‐di‐GMP‐bound Q190E and the Q218E mutants using the thermodynamic cycle shown in Figure 2a and the formula:

$$K_{D}=K_{b}\frac{1+10^{{\left(pK_{a}\right)}_{b}-pH}}{1+10^{{\left(pK_{a}\right)}_{f}-pH}}$$

It describes the experimentally measured K_D_ as a function of ligand binding to both the protonated and the unprotonated form and the pH.^[^ ^28^ ^]^ Fitting the K_D_’s measured over a pH range from 4.0 to 8.5 (Figure 2b, Table S3) to this formula yielded pK_a_ values of 7.5 and 6.3 for the Q190E/c‐di‐GMP complex and the free Q190E mutant protein, respectively. For the Q218E/c‐di‐GMP‐complex and the free Q218E protein, pK_a_ values of 7.1 and 5.9 were determined (Figure 2b). This shows that at physiologically relevant pH values of ∼ 7.0, the protonation of the glutamate side chain contributes significantly to ligand binding. pK_a_ values typically measured for glutamate carboxylate groups in unstructured peptides and on protein surfaces are on the order of ∼4.5–5.^[^ ^13^, ^29^ ^]^ In contrast, the pK_a_ values of E190 and E218 in the ligand free proteins are already significantly higher. This suggests an important role for the specific protein environment in the protonation‐mediated ligand binding process. The increased pK_a_ for the carboxylate group of residue E190 in the unliganded Q190E mutant is also in agreement with the Cδ chemical shift for the E190 side chain at pH 5.8 which is 179.9 ppm, a significant upfield shift compared to the Cδ chemical shifts of the other, unprotonated glutamate side chains^[^ ^26^ ^]^ (Figures 2c and S8).

In the WT protein, the two phosphodiester groups of c‐di‐GMP are also hydrogen bonded to the amino groups of the Q190 and Q218 side chains as well as to the backbone amide groups of L197 and L168, respectively^[^ ^22^ ^]^ (Figure 1b). Furthermore, the Q190 and Q218 side chain oxygen atoms form hydrogen bonds to the 2′‐OH groups of the ribose moieties (Figure 1b). Using NMR spectroscopy, we analyzed how this hydrogen bonding network is impacted by the replacement of the carboxamide group in glutamine with a protonated carboxylate group in the Q/E mutants.

**Figure 3:**
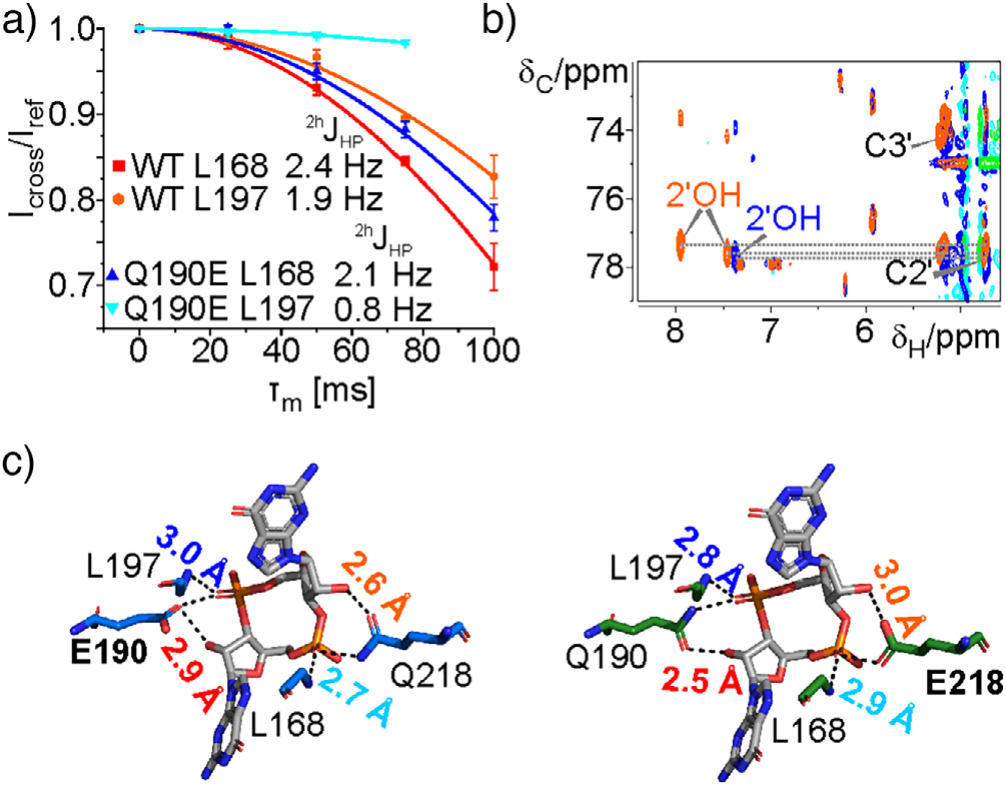
The intermolecular hydrogen bond network in the GSPII‐B domain phosphodiester binding pockets is modulated by the Q190E and Q218E substitutions. a) ^2h^J_HP_ cross‐hydrogen bond scalar couplings of the L168 amide and the L197 amide hydrogen bonds to the non‐bridging oxygen atoms of the phosphodiester groups of c‐di‐GMP. The scalar couplings of the ligand‐bound PilF_159‐221_ WT are shown in red (L168) and orange (L197) and for the ligand‐bound PilF_159‐221_ Q190E in blue (L168) and cyan (L197). b) Overlay of the 2D‐^13^C‐TOCSY‐HSQC spectra of ^13^C‐labelled c‐di‐GMP bound to PilF_159‐221_ WT (orange) and PilF_159‐221_ Q190E (blue). Correlations of the 2′‐OH signals to the C2′ resonances are indicated as grey dashed lines. c) Mutant and native phosphodiester binding pockets of Q190E (left) and Q218E (right) in complex with c‐di‐GMP. Hydrogen bonds are indicated as black dashed lines. Hydrogen bond lengths are shown in blue for L197, in cyan for L168, red for E190 (left), and Q190 and in orange for Q218 and E218 (right).

The ^15^N‐HSQC spectra for the WT and the Q190E subdomain bound to c‐di‐GMP are overall very similar but reveal a large chemical shift difference for the backbone amide group of L197 (Figure S6c). This backbone amide group forms a hydrogen bond to the same phosphodiester group as the E190 carboxylate group (Figure 1b). Since the L197 backbone amide group signal is shifted upfield by ∼ 1.0 ppm in the Q190E mutant compared to the WT (Figure S6c), this suggests a significant weakening of the respective intermolecular hydrogen bond to the ribose. A quantitative measurement of the ^2h^J_HP_ cross‐hydrogen bond scalar coupling between the backbone amide proton and the ^31^P nucleus^[^ ^30^, ^31^ ^]^ of the phosphodiester group also showed a reduction of the coupling constant from 1.9 Hz in the WT to 0.8 Hz in the mutant in agreement with a significant lengthening of this hydrogen bond (Figure 3a). In contrast, the coupling constant between the backbone amide proton of the second leucine residue, L168, and the phosphorous nucleus in the unaltered ligand binding site is similar between WT and mutant (2.4 vs. 2.1 Hz).

Hydrogen bonding protects the 2′‐OH protons from exchange with the solvent. Accordingly, the corresponding NMR signals for these protons can be detected in ^13^C‐edited TOCSY‐HSQC experiments^[^ ^32^ ^]^ with ^13^C‐labeled c‐di‐GMP bound to unlabeled protein (Figure 3b). This allows to qualitatively characterize the hydrogen bonds between the 2′‐OH groups of the ribose moieties from c‐di‐GMP and the side chains of protein residues 190 and 218. In the ^13^C‐TOCSY‐HSQC of the WT protein, two ribose 2′‐OH signals are observable for the two ribose units of the bound c‐di‐GMP ligand (Figure 3b). In contrast, the signal corresponding to the 2′‐OH proton of the ribose hydrogen bonded to the E190 side chain is no longer visible showing that this hydrogen bond is weakened in the mutant. These results agree with the hydrogen bond length measurements in our crystal structures of the two mutants (Figure 3c), where we observed that the hydrogen bond between the L197 amide group and the non‐bridging oxygen of the phosphodiester group is 0.2 Å longer in the Q190E construct than the same hydrogen bond in the Q218E mutant, where this phosphodiester binding pocket is in its WT configuration. Similarly, the hydrogen bond length between the L168 amide group and the phosphodiester oxygen in the Q218E mutant is increased by 0.2 Å compared to the native binding site in the Q190E construct (Figure 3c, d, orange numbers). Finally, the lengths of the hydrogen bonds from E190 to the 2′‐OH group of c‐di‐GMP in the Q190E mutant and from E218 to the 2′‐OH group in the Q218E mutant are increased by 0.4 Å if compared to the native sites in both constructs (Figure 3c, d, red numbers). Together, the NMR data and X‐ray structures suggest that upon substitution of the native glutamine with glutamate, the hydrogen bonding network in the c‐di‐GMP binding site of the GSPII‐B domain of PilF is adjusted towards favoring a short hydrogen bond between the protonated oxygen in the carboxylate group of the glutamate and the phosphodiester at the expense of the other two intermolecular hydrogen bonds in the hydrogen bond network.

Our detailed characterization of the interaction of the GSPII‐B domain Q‐to‐E mutants with the phosphodiester groups of c‐di‐GMP indicated that the carboxylate groups of the relevant acidic amino acid side chains are readily protonated at physiologically relevant pH values and then able to act as hydrogen bond donor groups thereby contributing favorably to ligand affinity. A decisive factor for the importance of such interactions is the specific structural environment of the respective carboxylate group bearing side chains since it likely contributes significantly to shifts of their pK_a_’s toward neutrality. We therefore wondered whether the potential of acidic sidechains as a general binding motif for phosphodiester groups may have been overlooked so far in previous structural studies of protein‐nucleic acid interactions.

**Figure 4:**
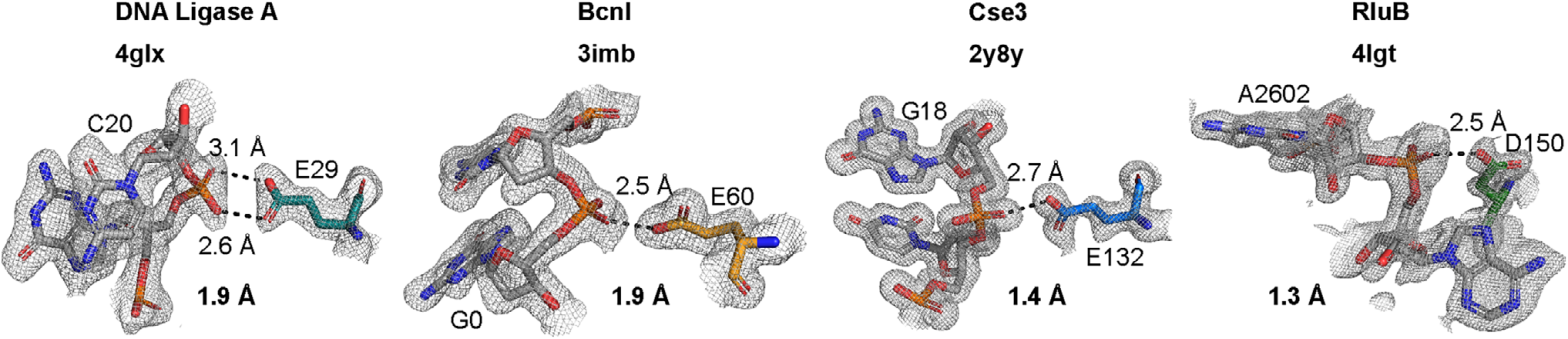
Examples of glutamate/aspartate carboxylate groups in phosphodiester group recognition by DNA/RNA binding proteins. Shown are select examples of crystal structures for a DNA ligase (PDB: 4GLX),^[^ ^33^ ^]^ a restriction endonuclease (PDB: 3IMB),^[^ ^34^ ^]^ an endoribonuclease (PDB: 2Y8Y),^[^ ^35^ ^]^ and a pseudouridine synthase (PDB: 4LGT)^[^ ^36^ ^]^ in complex with a DNA or RNA ligand. For each example, the protein name, the respective pdb code, and the obtained resolution are given. Hydrogen bonds are indicated as black dashed lines and electron density is shown as a grey mesh at a σ level of 1.0.

To elucidate whether the interaction of phosphodiester groups with protonated glutamate and aspartate side chains could be a more general feature of biomacromolecular interactions, we looked at all entries of the protein data bank (PDB, www.rcsb.org) containing X‐ray structures of RNA‐protein or DNA‐protein complexes with a resolution better than 2.8 Å. The atomic coordinates were used to identify entries with distances smaller than 3.0 Å between the Oγ and Oδ oxygen atoms of aspartates and glutamates, respectively, with the OP1 and OP2 non‐bridging oxygen atoms of phosphodiester groups. Since this initial list contained many entries of RNA/DNA‐polymerases, nucleases, gyrases, ligases, and other enzymes acting on nucleic acids which often contain aspartate and glutamate residues complexed to divalent metal ions in their active centers which are naturally in close proximity to the phosphodiester backbone due to their catalytic activities, we excluded such examples from further analysis upon visual inspection of the respective entries. In addition, entries of complexes crystallized at pH values below the pK_a_ value of glutamate carboxylate groups in unstructured peptides (∼ 4.4, the pK_a_ value for aspartate carboxylate groups in unstructured peptides is ∼ 3.9^[^ ^12^, ^13^, ^14^ ^]^) were excluded. Nonetheless, numerous protein nucleic acid complexes featuring glutamate and aspartate side chain carboxylate groups oriented relative to phosphodiester groups in a fashion strongly suggestive of the formation of hydrogen bond interactions between protonated carboxylate groups and the non‐bridging oxygen atoms of the phosphodiester groups were identified. Examples come from different functional protein categories including, e.g., DNA ligases, restriction enzymes and RNA modification enzymes and cover proteins interacting both with DNA and RNA (Figures 4 and S9).

## Conclusion

According to our results presented here, hydrogen bonding interactions with protonated carboxylate groups of acidic amino acid side chains serving as hydrogen bond donors should be more widely considered when analyzing nucleic acid and cyclic (di‐) nucleotide binding motifs in proteins. Particular care should be taken when interpreting the results of mutation experiments where polar and positively charged amino acid side chains are replaced by glutamate and aspartate residues. In summary, our work shows that the interaction of phosphodiesters with the carboxylate groups of the acidic amino acid side chains in their protonated states seem to be a hitherto underappreciated feature in protein nucleic acid interactions.

## Supporting Information

The experimental methods and supplementary data (Tables S1‐S3 and Figures S1–S9) are available in the Supporting Information. The authors have cited additional references in the Supporting Information.^[^ ^26^, ^27^, ^37^, ^38^, ^39^, ^40^, ^41^, ^42^, ^43^, ^44^, ^45^, ^46^, ^47^, ^48^, ^49^, ^50^, ^51^, ^52^, ^53^, ^54^, ^55^, ^56^, ^57^ ^]^

## Conflict of Interests

The authors declare no conflict of interest.

## Supporting information



Supporting Information

## Data Availability

The data have been submitted to the PDB under accession codes 9GLG and 9GL5 and are on “hold until publication” as is common practice in structural biology.

## References

[1] P. Thaplyal , P. C. Bevilacqua , Methods Enzymol 2014, 549, 189–219. 25432750 10.1016/B978-0-12-801122-5.00009-X PMC5597436

[2] K. Nadassy , S. J. Wodak , J. Janin , Biochemistry 1999, 38, 1999–2017. 10026283 10.1021/bi982362d

[3] S. A. Coulocheri , D. G. Pigis , K. A. Papavassiliou , A. G. Papavassiliou , Biochimie 2007, 89, 1291–1303. 17825469 10.1016/j.biochi.2007.07.020

[4] S. Jones , P. van Heyningen , H. M. Berman , J. M. Thornton , J. Mol. Biol. 1999, 287, 877–896. 10222198 10.1006/jmbi.1999.2659

[5] R. P. Bahadur , M. Zacharias , J. Janin , Nucleic Acids Res. 2008, 36, 2705–2716. 18353859 10.1093/nar/gkn102 PMC2377425

[6] M. Treger , E. Westhof , J. Mol. Recog. 2001, 14, 199–214. 10.1002/jmr.534 11500966

[7] S. Jones , D. T. A. Daley , N. M. Luscombe , H. M. Berman , J. M. Thornton , Nucleic Acids Res. 2001, 29, 943–954. 11160927 10.1093/nar/29.4.943 PMC29619

[8] X. Zeng , M. Huang , Q.‐X. Sun , Y.‐J. Peng , X. Xu , Y.‐B. Tang , J.‐Y. Zhang , Y. Yang , C.‐C. Zhang , Proc. Natl. Acad. Sci. USA 2023, 120, e2221874120. 36947515 10.1073/pnas.2221874120 PMC10068817

[9] N. M. Luscombe , S. E. Austin , H. M. Berman , J. M. Thornton , Genome Biol. 2000, 1, reviews001.1–reviews001.37. 10.1186/gb-2000-1-1-reviews001 PMC138832 11104519

[10] D. E. Draper , Annu. Rev. Biochem. 1995, 64, 593–620. 7574494 10.1146/annurev.bi.64.070195.003113

[11] D. E. Draper , J. Mol. Biol. 1999, 293, 255–270. 10550207 10.1006/jmbi.1999.2991

[12] Y. Nozaki , C. Tanford , J. Biol. Chem. 1967, 242, 4731–4735. 6061418

[13] M. Tollinger , J. D. Forman‐Kay , L. E. Kay , J. Am. Chem. Soc. 2002, 124, 5714–5717. 12010044 10.1021/ja020066p

[14] P. Keim , R. A. Vigna , J. S. Morrow , R. C. Marshall , F. R. N. Gurd , J. Biol. Chem. 1973, 248, 7811–7818. 4750428

[15] R. Simm , M. Morr , A. Kader , M. Nimtz , U. Römling , Mol. Microbiol. 2004, 53, 1123–1134. 15306016 10.1111/j.1365-2958.2004.04206.x

[16] A. J. Wolfe , K. L. Visick , J. Bacteriol. 2008, 190, 463–475. 17993515 10.1128/JB.01418-07 PMC2223684

[17] C. R. Guzzo , R. K. Salinas , M. O. Andrade , C. S. Farah , J. Mol. Biol. 2009, 393, 848–866. 19646999 10.1016/j.jmb.2009.07.065

[18] R. Tamayo , J. T. Pratt , A. Camilli , Annu. Rev. Microbiol. 2007, 61, 131–148. 17480182 10.1146/annurev.micro.61.080706.093426 PMC2776827

[19] R. Hengge , Nat. Rev. Microbiol. 2009, 7, 263–273. 19287449 10.1038/nrmicro2109

[20] R. Salzer , F. Joos , B. Averhoff , Appl. Environ. Microbiol. 2014, 80, 644–652. 24212586 10.1128/AEM.03218-13 PMC3911100

[21] K. Kruse , R. Salzer , F. Joos , B. Averhoff , Extremophiles 2018, 22, 461–471. 29464394 10.1007/s00792-018-1008-9

[22] K. Neißner , H. Keller , L. Kirchner , S. Düsterhus , E. Duchardt‐Ferner , B. Averhoff , J. Wöhnert , J. Biol. Chem. 2025, 301, 108041. 39615687 10.1016/j.jbc.2024.108041 PMC11731258

[23] Y.‐C. Wang , K.‐H. Chin , Z.‐L. Tu , J. He , C. J. Jones , D. Z. Sanchez , F. H. Yildiz , M. Y. Galperin , S.‐H. Chou , Nat. Commun. 2016, 7, 12481. 27578558 10.1038/ncomms12481 PMC5013675

[24] J. C.‐H. Chen , B. L. Hanson , S. Z. Fisher , P. Langan , A. Y. Kovalevsky , Proc. Natl. Acad. Sci. USA 2012, 109, 15301–15306. 22949690 10.1073/pnas.1208341109 PMC3458323

[25] M. A. S. Hass , F. A. A. Mulder , Ann Rev Biophys 2015, 44, 53–75. 25747592 10.1146/annurev-biophys-083012-130351

[26] M. Betz , F. Löhr , H. Wienk , H. Rüterjans , Biochemistry 2004, 43, 5820–5831. 15134456 10.1021/bi049948m

[27] M. Pellecchia , H. Iwai , T. Szyperski , K. Wüthrich , J. Magn. Reson. 1997, 124, 274–278. 9424317 10.1006/jmre.1996.1058

[28] J. M. Bradshaw , G. Waksman , Biochemistry 1998, 37, 15400–15407. 9799501 10.1021/bi9814991

[29] W. R. Forsyth , J. M. Antosiewicz , A. D. Robertson , Proteins 2002, 48, 388–403. 12112705 10.1002/prot.10174

[30] E. Duchardt‐Ferner , J. Ferner , J. Wöhnert , Angew Chem Int Ed Engl 2011, 50, 7927–7930. 21837618 10.1002/anie.201101743

[31] E. Duchardt‐Ferner , J. Wöhnert , J. Biomol. NMR 2017, 69, 101–110. 29032519 10.1007/s10858-017-0140-7

[32] M. Hennig , J. Fohrer , T. Carlomagno , J. Am. Chem. Soc. 2005, 127, 2028–2029. 15713064 10.1021/ja043390o

[33] J.‐P. Surivet , R. Lange , C. Hubschwerlen , W. Keck , J.‐L. Specklin , D. Ritz , D. Bur , H. Locher , P. Seiler , D. S. Strasser , L. Prade , C. Kohl , C. Schmitt , G. Chapoux , E. Ilhan , N. Ekambaram , A. Athanasiou , A. Knezevic , D. Sabato , A. Chambovey , M. Gaertner , M. Enderlin , M. Boehme , V. Sippel , P. Wyss , Bioorg. Med. Chem. Lett. 2012, 22, 6705–6711. 23006603 10.1016/j.bmcl.2012.08.094

[34] M. Sokolowska , M. Kaus‐Drobek , H. Czapinska , G. Tamulaitis , R. H. Szczepanowski , C. Urbanke , V. Siksnys , M. Bochtler , J. Mol. Biol. 2007, 369, 722–734. 17445830 10.1016/j.jmb.2007.03.018

[35] D. G. Sashital , M. Jinek , J. A. Doudna , Nat. Struct. Mol. Biol. 2011, 18, 680–687. 21572442 10.1038/nsmb.2043

[36] N. Czudnochowski , G. W. Ashley , D. V. Santi , A. Alian , J. Finer‐Moore , R. M. Stroud , Nucleic Acids Res. 2014, 42, 2037–2048. 24214967 10.1093/nar/gkt1050 PMC3919597

[37] E. Mejia , M. Burak , A. Alonso , V. Larraga , T. A. Kunkel , K. Bebenek , M. Garcia‐Diaz , DNA Repair 2014, 18, 1–9. 24666693 10.1016/j.dnarep.2014.03.001 PMC4040948

[38] S.‐J. Choi , C. Ban , Sci. Rep. 2016, 11, 34998. 10.1038/srep34998 PMC5057103 27725738

[39] L. Cheng , F. Li , Y. Jiang , H. Yu , C. Xie , Y. Shi , Q. Gong , Nucleic Acids Res. 2018, 47, 495–508. 10.1093/nar/gky1116 PMC6326804 30407553

[40] H. Hashimoto , Y. O. Olanrewaju , Y. Zheng , G. G. Wilson , X. Zhang , X. Cheng , Genes Dev. 2014, 28, 2304–2313. 25258363 10.1101/gad.250746.114 PMC4201290

[41] O. Edelheit , A. Hanukoglu , I. Hanukoglu , BMC Biotechnol. 2009, 9, 61. 19566935 10.1186/1472-6750-9-61 PMC2711942

[42] D. G. Gibson , L. Young , R.‐Y. Chuang , J. C. Venter , C. A. Hutchison , H. O. Smith , Nat. Methods 2009, 6, 343–345. 19363495 10.1038/nmeth.1318

[43] K. Neißner , H. Keller , E. Duchardt‐Ferner , C. Hacker , K. Kruse , B. Averhoff , J. Wöhnert , Biomol NMR Assign 2019, 13, 383–390. 31432400 10.1007/s12104-019-09911-z

[44] F. Rao , S. Pasunooti , Y. Ng , W. Zhuo , L. Lim , A. W. Liu , Z.‐X. Liang , Anal. Biochem. 2009, 389, 138–142. 19328769 10.1016/j.ab.2009.03.031

[45] H. Keller , A. K. Weickhmann , T. Bock , J. Wöhnert , RNA 2018, 24, 1390–1402. 30006500 10.1261/rna.067470.118 PMC6140456

[46] W. Kabsch , Acta Cryst D 2010, 66, 133–144. 20124693 10.1107/S0907444909047374 PMC2815666

[47] P. Evans , Acta Cryst D 2006, 62, 72–82. 16369096 10.1107/S0907444905036693

[48] P. R. Evans , Acta Cryst D 2011, 67, 282–292. 21460446 10.1107/S090744491003982X PMC3069743

[49] D. Liebschner , P. V. Afonine , M. L. Baker , G. Bunkóczi , V. B. Chen , T. I. Croll , B. Hintze , L.‐W. Hung , S. Jain , A. J. McCoy , N. W. Moriarty , R. D. Oeffner , B. K. Poon , M. G. Prisant , R. J. Read , J. S. Richardson , D. C. Richardson , M. D. Sammito , O. V. Sobolev , D. H. Stockwell , T. C. Terwilliger , A. G. Urzhumtsev , L. L. Videau , C. J. Williams , P. D. Adams , Acta Cryst D 2019, 75, 861–877. 10.1107/S2059798319011471 PMC6778852 31588918

[50] P. V. Afonine , R. W. Grosse‐Kunstleve , N. Echols , J. J. Headd , N. W. Moriarty , M. Mustyakimov , T. C. Terwilliger , A. Urzhumtsev , P. H. Zwart , P. D. Adams , Acta Cryst D 2012, 68, 352–367. 22505256 10.1107/S0907444912001308 PMC3322595

[51] C. J. Williams , J. J. Headd , N. W. Moriarty , M. G. Prisant , L. L. Videau , L. N. Deis , V. Verma , D. A. Keedy , B. J. Hintze , V. B. Chen , S. Jain , S. M. Lewis , W. B. Arendall , J. Snoeyink , P. D. Adams , S. C. Lovell , J. S. Richardson , D. C. Richardson , Protein Sci. 2018, 27, 293–315. 29067766 10.1002/pro.3330 PMC5734394

[52] J. L. Markley , A. Bax , Y. Arata , C. W. Hilbers , R. Kaptein , B. D. Sykes , P. E. Wright , K. Wüthrich , J. Mol. Biol. 1998, 70, 117–142. 10.1006/jmbi.1998.1852 9671561

[53] R. Powers , A. M. Gronenborn , G. Marius Clore , A. Bax , J. Magn. Reson. 1991, 94, 209–213.

[54] S. Grzesiek , A. Bax , J Magn Reson Series B 1993, 102, 103–106.

[55] S. Raschka , JOSS 2017, 2, 279.

[56] M. W. Maciejewski , A. D. Schuyler , M. R. Gryk , I. I. Moraru , P. R. Romero , E. L. Ulrich , H. R. Eghbalnia , M. Livny , F. Delaglio , J. C. Hoch , Biophys. J. 2017, 112, 1529–1534. 28445744 10.1016/j.bpj.2017.03.011 PMC5406371

[57] K. Baskaran , D. L. Craft , H. R. Eghbalnia , M. R. Gryk , J. C. Hoch , M. W. Maciejewski , A. D. Schuyler , J. R. Wedell , C. W. Wilburn , Front. Mol. Biosci. 2022, 8, 817175. 35111815 10.3389/fmolb.2021.817175 PMC8802229

