# Supplementary material for: Protonated Glutamate and Aspartate Side Chains Can Recognize Phosphodiester Groups via Strong and Short Hydrogen Bonds in Biomacromolecular Complexes
Source: Angew Chem Int Ed Engl. 2025 May 30;64(27):e202501589. doi: 10.1002/anie.202501589 (PMC12207366; doi:10.1002/anie.202501589)
Supplement: Supplementary file 1 — Supporting Information [file ANIE-64-e202501589-s001.docx]

**Supporting Information**

**Protonated Glutamate and Aspartate Side Chains Can Recognize Phosphodiester Groups via Strong and Short Hydrogen Bonds in Biomacromolecular Complexes**

Konstantin Neissner^1,2^, Elke Duchardt-Ferner^1,2^, Christoph Wiedemann^3^, Julian Kraus^1^, Ute A. Hellmich^2,3,4^, Jens Wöhnert^1,2*^

^1^Institute for Molecular Biosciences, Goethe-University Frankfurt/M., Max-von-Laue-Str. 9, 60438 Frankfurt, Germany

^2^Center for Biomolecular Magnetic Resonance (BMRZ), Goethe-University Frankfurt/M., Max-von-Laue-Str. 9, 60438 Frankfurt, Germany

^3^Institute of Organic Chemistry and Macromolecular Chemistry (IOMC), Friedrich-Schiller-University Jena, Humboldtstraße 10, 07743 Jena, Germany

^4^Cluster of Excellence “Balance of the Microverse”, Friedrich-Schiller-University Jena, 07743 Jena, Germany

*To whom correspondence should be addressed:

woehnert@bio.uni-frankfurt.de

**Supplementary Tables**

**Table S1: Statistics of data collection and refinement for X-ray datasets**

|  | **PilF_159-302_ Q190E** | **PilF_159-302_ Q218E** |
| --- | --- | --- |
| **Data collection** |  |  |
| Wavelength (Å) | 0.976 | 0.976 |
| Resolution range | 32.71 – 1.9 (1.968 – 1.9) | 39.14 - 1.55 (1.605 - 1.55) |
| Space group | R 3 :H | R 3 :H |
| Unit cell | 107.966 107.966 86.366 90 90 120 | 108.099 108.099 86.183 90 90 120 |
| Total reflections | 306406 (31593) | 562192 (56474) |
| Unique reflections | 29580 (2977) | 54531 (5482) |
| Multiplicity | 10.4 (10.6) | 10.3 (10.3) |
| Completeness (%) | 99.97 (100.00) | 99.98 (100.00) |
| Mean I/sigma(I) | 10.12 (0.97) | 12.78 (1.01) |
| Wilson B-factor | 44.91 | 31.16 |
| R-merge | 0.1055 (1.871) | 0.08074 (1.838) |
| R-meas | 0.1109 (1.967) | 0.08503 (1.934) |
| R-pim | 0.034 (0.6034) | 0.02645 (0.5994) |
| CC1/2 | 0.997 (0.636) | 0.997 (0.594) |
| CC* | 0.999 (0.882) | 0.999 (0.863) |
| **Refinement** |  |  |
| Reflections used | 29574 (2977) | 54526 (5482) |
| Reflections used for R-free | 1622 (165) | 1771 (180) |
| R-work | 0.1928 | 0.1863 |
| R-free | 0.2242 | 0.2083 |
| CC(work) | 0.967 | 0.961 |
| CC(free) | 0.957 | 0.957 |
| Number of non-hydrogen atoms | 2376 | 2580 |
| macromolecules | 2116 | 2158 |
| ligands | 138 | 135 |
| solvent | 122 | 287 |
| Protein residues | 276 | 277 |
| RMS(bonds) | 0.006 | 0.007 |
| RMS(angles) | 0.79 | 0.87 |
| Ramachandran favored (%) | 100.00 | 100.00 |
| Ramachandran allowed (%) | 0.00 | 0.00 |
| Ramachandran outliers (%) | 0.00 | 0.00 |
| Rotamer outliers (%) | 0.00 | 0.00 |
| Clashscore | 3.84 | 4.45 |
| Average B-factor | 58.77 | 42.00 |
| macromolecules | 58.10 | 40.18 |
| ligands | 67.00 | 49.34 |
| solvent | 60.98 | 52.26 |
| Number of TLS groups | 12 | 11 |
| **PDB codes** | 9GL5 | 9GLG |

**Table S2: Overview of pairwise RMSD values for the backbone heavy atoms of PilF_159-302_ WT (PDB entry 8pdk) and the respective Q190E and Q218E mutants.**

| **RMSD** | **PilF_159-302_ WT** | **Q190E** | **Q218E** |
| --- | --- | --- | --- |
| **PilF_159-302_ WT** | / | 0.15 Å | 0.16 Å |
| **Q190E** | 0.15 Å | / | 0.14 Å |
| **Q218E** | 0.16 Å | 0.14 Å | / |

**Table S3:** **ITC data for c-di-GMP binding to PilF_159-302_ constructs**

| **Construct** | **Sample buffer** | **Temperature**  **[°C]** | **pH** | **N** | **K_D_**  **[nM]** | **ΔH**  **[kcal/mol]** | **-TΔS**  **[kcal/mol]** | **ΔG**  **[kcal/mol]** |
| --- | --- | --- | --- | --- | --- | --- | --- | --- |
| **PilF_159-302_ WT** | Tris | 20 | 8.5 | 0.74 | 9 | -11.41 | 0.63 | -10.78 |
| **PilF_159-302_ WT** | Na-Acetate | 20 | 4.0 | 0.99 | 4 | -6.45 | -4.81 | -11.26 |
| **PilF_159-302_ Q190E** | Tris | 20 | 8.5 | 0.86 ± 0.09 | 798 ± 39 | -5.66 ± 0.17 | -2.52 ± 0.20 | -8.18 ± 0.03 |
| **PilF_159-302_ Q190E** | Tris | 20 | 8.0 | 0.84 ± 0.17 | 729 ± 44 | -4.87 ± 0.68 | -3.37 ± 0.65 | -8.23 ± 0.04 |
| **PilF_159-302_ Q190E** | Tris | 20 | 7.5 | 0.95 ± 0.09 | 511 ± 13 | -3.81 ± 0.20 | -4.63 ± 0.19 | -8.44 ± 0.02 |
| **PilF_159-302_ Q190E** | Bis-Tris | 20 | 7.0 | 0.98 ± 0.01 | 236 ± 8 | -4.76 ± 0.21 | -4.14 ± 0.23 | -8.88 ± 0.02 |
| **PilF_159-302_ Q190E** | Bis-Tris | 20 | 6.5 | 0.72 ± 0.12 | 160 ± 34 | -5.58 ± 0.09 | -3.55 ± 0.20 | -9.12 ± 0.13 |
| **PilF_159-302_ Q190E** | Bis-Tris | 20 | 6.0 | 0.96 ± 0.01 | 67 ± 3 | -5.97 ± 0.05 | -3.45 ± 0.05 | -9.40 ± 0.02 |
| **PilF_159-302_ Q190E** | Na-Acetate | 20 | 5.5 | 1.02 ± 0.02 | 74 ± 8 | -8.54 ± 0.04 | -1.11 ± 0.16 | -9.56 ± 0.07 |
| **PilF_159-302_ Q190E** | Na-Acetate | 20 | 5.0 | 0.91 ± 0.04 | 78 ± 11 | -9.28 ± 0.43 | -0.27 ± 0.51 | -9.54 ± 0.09 |
| **PilF_159-302_ Q190E** | Na-Acetate | 20 | 4.5 | 0.95 ± 0.13 | 42 ± 13 | -7.65 ± 0.98 | -2.27 ± 0.87 | -9.92 ± 0.18 |
| **PilF_159-302_ Q190E** | Na-Acetate | 20 | 4.0 | 0.98 ± 0.01 | 40 ± 13 | -7.37 ± 0.17 | -2.59 ± 0.37 | -9.96 ± 0.22 |
| **PilF_159-302_ Q190E** | Tris | 20 | 7.0 | 0.58 | 223 | -2.81 | -6.09 | -8.92 |
| **PilF_159-302_ Q190E** | Bis-Tris | 20 | 7.5 | 0.96 | 505 | -5.93 | -2.51 | -8.44 |
| **PilF_159-302_ Q190E** | Bis-Tris | 20 | 5.5 | 0.89 | 71 | -7.06 | -2.53 | -9.58 |
| **PilF_159-302_ Q190E** | Na-Acetate | 20 | 6.0 | 0.86 | 96 | -8.93 | -0.49 | -9.41 |
| **PilF_159-302_ Q218E** | Tris | 20 | 8.5 | 0.73 ± 0.14 | 463 ± 13 | -10.65 ± 0.16 | 2.15 ± 0.18 | -8.49 ± 0.02 |
| **PilF_159-302_ Q218E** | Tris | 20 | 8.0 | 1.12 ± 0.05 | 432 ± 25 | -9.52 ± 0.31 | 0.99 ± 0.27 | -8.53 ± 0.03 |
| **PilF_159-302_ Q218E** | Tris | 20 | 7.5 | 0.92 ± 0.07 | 342 ± 26 | -5.45 ± 0.13 | -3.22 ± 0.17 | -8.67 ± 0.04 |
| **PilF_159-302_ Q218E** | Bis-Tris | 20 | 7.0 | 0.90 ± 0.16 | 249 ± 7 | -6.80 ± 0.08 | -2.05 ± 0.07 | -8.85 ± 0.02 |
| **PilF_159-302_ Q218E** | Bis-Tris | 20 | 6.5 | 1.00 ± 0.27 | 110 ± 3 | -7.86 ± 0.22 | -1.48 ± 0.23 | -9.33 ± 0.02 |
| **PilF_159-302_ Q218E** | Bis-Tris | 20 | 6.0 | 1.03 ± 0.23 | 64 ± 7 | -7.42 ± 0.75 | -2.23 ± 0.80 | -9.64 ± 0.06 |
| **PilF_159-302_ Q218E** | Na-Acetate | 20 | 5.5 | 0.97 ± 0.09 | 28 ± 10 | -9.78 ± 0.92 | -0.40 ± 1.00 | -10.17 ± 0.20 |
| **PilF_159-302_ Q218E** | Na-Acetate | 20 | 5.0 | 1.31 ± 0.09 | 36 ± 8 | -8.53 ± 0.44 | -1.47 ± 0.47 | -9.99 ± 0.13 |
| **PilF_159-302_ Q218E** | Na-Acetate | 20 | 4.5 | 0.90 ± 0.04 | 61 ± 7 | -7.12 ± 0.44 | -2.56 ± 0.49 | -9.68 ± 0.07 |
| **PilF_159-302_ Q218E** | Na-Acetate | 20 | 4.0 | 0.84 ± 0.07 | 23 ± 10 | -3.89 ± 1.80 | -2.37 ± 3.86 | -10.28 ± 0.24 |

**Supplementary Figures**

**
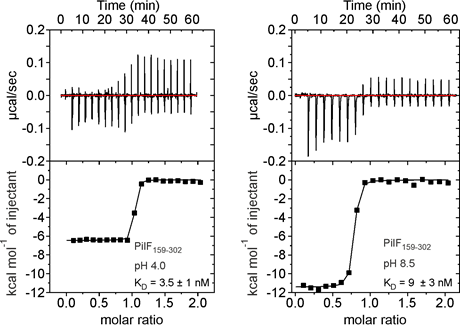
**

**Figure S1: Binding of c-di-GMP to wild-type PilF_159-302_ is pH independent.** ITC based c-di-GMP binding assay for WT PilF_159-302_ at pH 4.0 (left) and pH 8.5 (right).


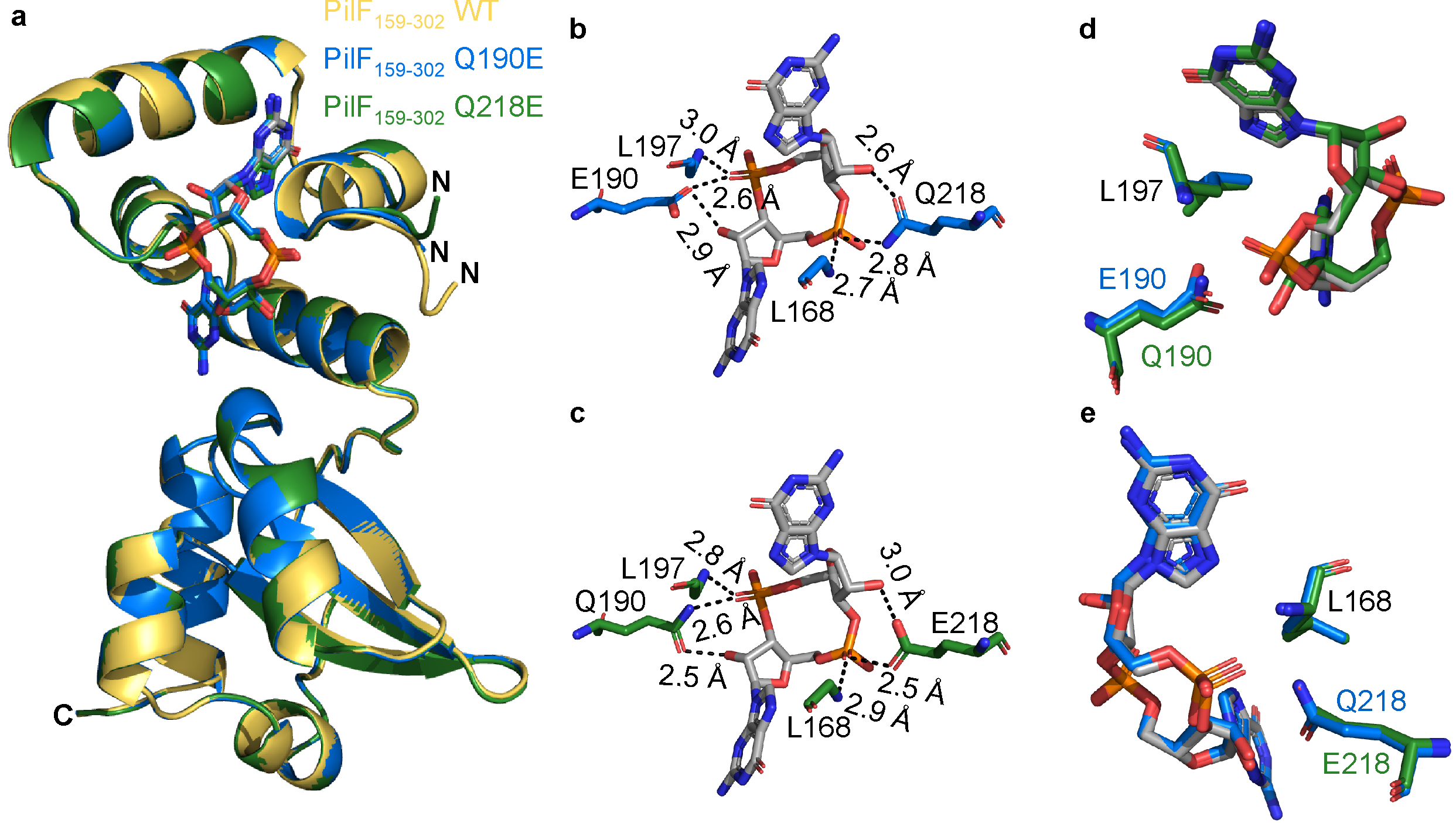


**Figure S2: Structural comparison of PilF_159-302_ variants in complex with c-di-GMP.** (a) Global superposition of the PilF_159-302_ Q190E and Q218E mutants in complex with c-di-GMP to WT PilF_159-302_ in complex with c-di-GMP (PDB-entry 8pdk ^[22]^, see Table S2). (b, c) c-di-GMP phosphodiester group recognition of PilF_159-302_ Q190E (b) and Q218E (c). Amino acid residues involved in intermolecular hydrogen bonding and the ligand are shown as sticks. c-di-GMP carbon atoms are shown in gray and the carbon atoms of the amino acids involved in ligand binding in marine blue (b) or green (c). Nitrogen atoms are colored blue, oxygen atoms red and phosphorous atoms orange. Hydrogen bonds are depicted as black dashed lines. (d) Superposition of the phosphodiester binding pockets involving E190 from the Q190E mutant and Q190 from the Q218E mutant. Carbon atoms of c-di-GMP bound by the Q218E construct are colored green to distinguish the c-di-GMP molecules. Otherwise, atoms are color coded as in (b) and (c). (e) Superposition of the phosphodiester binding pockets from the Q190E and Q218E mutants involving Q218 from the Q190E mutant and E218 from the Q218E mutant. Atoms are colored as above except for c-di-GMP complexed with the Q19OE construct, in which the carbon atoms are colored marine blue to distinguish this c-di-GMP from the c-di-GMP bound by Q218E.


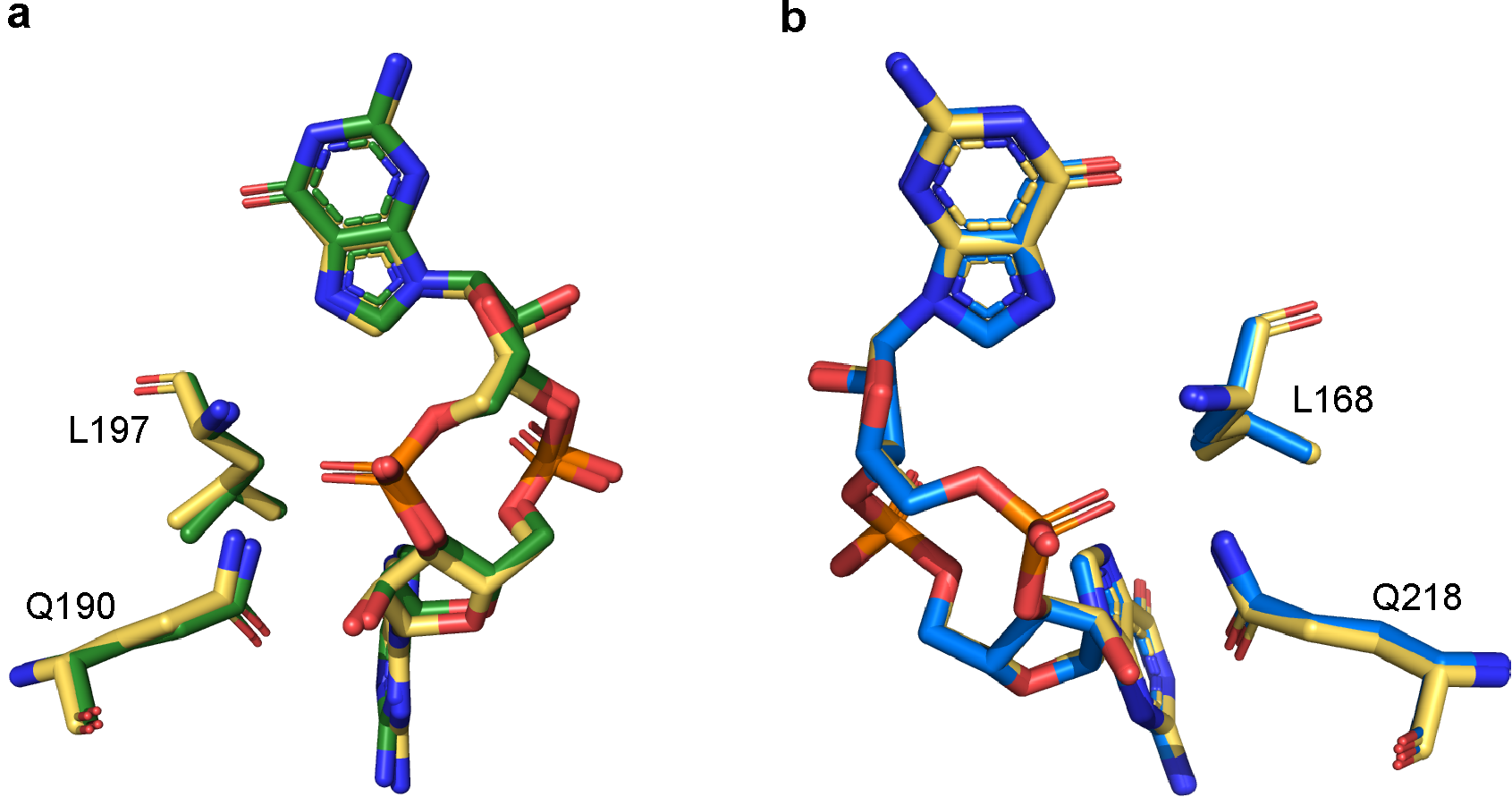


**Figure S3: Comparison of the native-like phosphodiester binding pockets of the PilF_159-302_ Q190E and Q218E constructs with PilF_159-302_ WT.** (a) Superposition of the native-like (Q190) phosphodiester binding pocket of Q218E and WT (RMSD 0.14 Å). Nitrogen atoms are colored blue, oxygen atoms are red and phosphorous atoms are orange. Carbon atoms of the Q218E residues are green and WT carbon atoms are yellow. Carbon atoms of c-di-GMP bound by WT are yellow. (b) Superposition of the native-like (Q218) phosphodiester binding pocket of Q190E and WT (RMSD 0.15 Å). Nitrogen atoms are colored blue, oxygen atoms are red and phosphorous atoms are orange. Carbon atoms of the Q190E residues are marine blue and WT carbon atoms are yellow. Carbon atoms of c-di-GMP bound by WT are yellow.


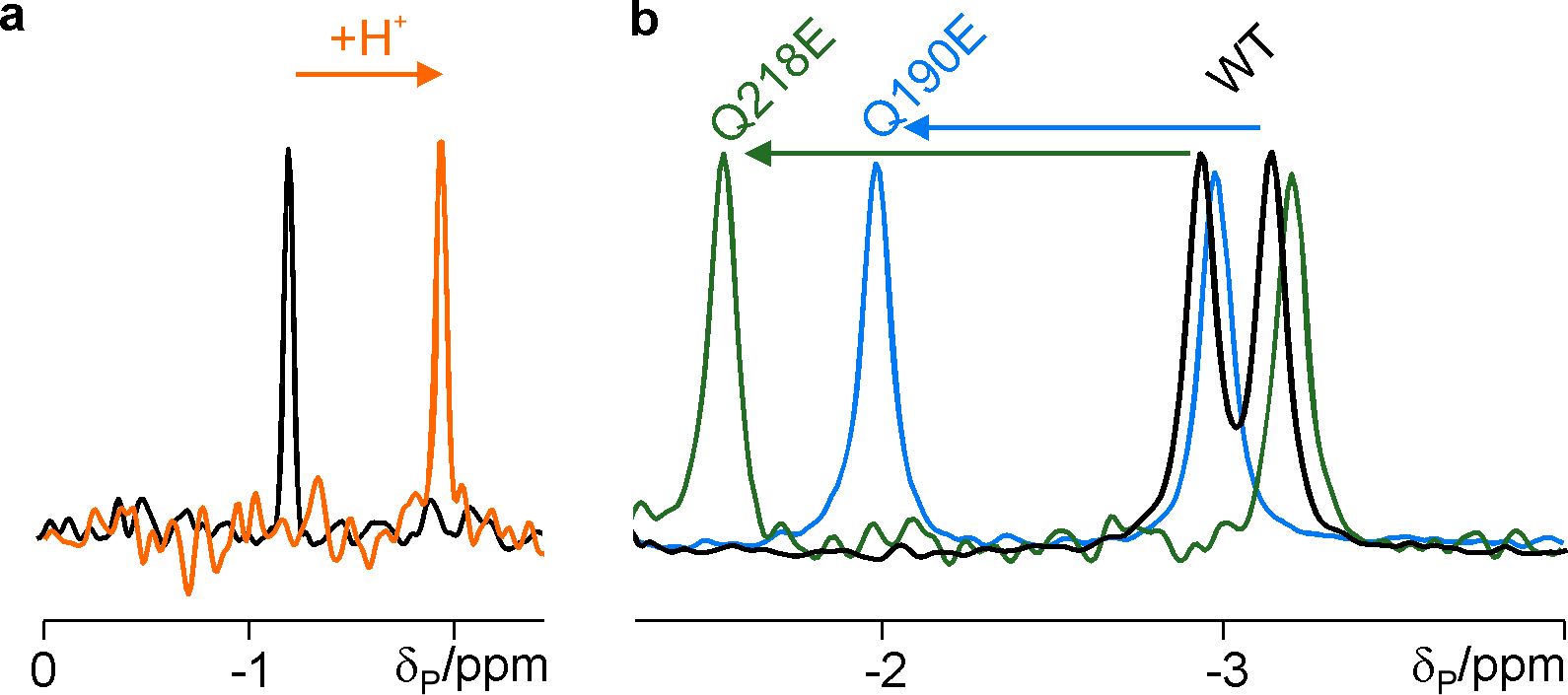


**Figure S4: Phosphodiester group protonation state of c-di-GMP**. (a) Overlay of 1D-^31^P spectra of free c-di-GMP in water pH 7.0 (black) and with 100 mM HCl (pH 1.0, orange). Protonation of the phosphodiester groups leads to an upfield change of the chemical shift. (b) Overlay of 1D-^31^P spectra of c-di-GMP complexed with PilF_159-302_ WT (black), Q190E (blue) and Q218E (green). Assignments for the ^31^P-signals of c-di-GMP bound to the WT protein are based on the results described previously ^[22]^.


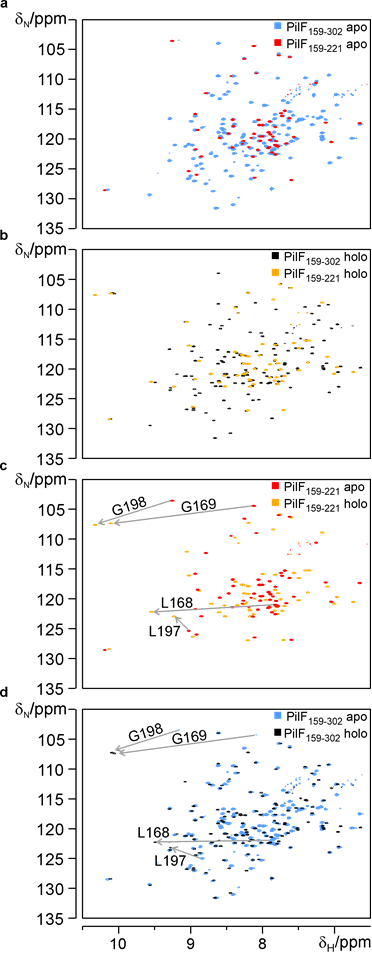


**Figure S5: Comparison of the ^15^N-BEST-TROSY-HSQC spectra of PilF_159-221_ WT to PilF_159-302_ WT in the free and the c-di-GMP-bound state**. (a) Overlay of the ^15^N-BEST-TROSY-HSQC spectra of free PilF_159-221_ WT on free PilF_159-302_ WT. (b) Overlay of the ^15^N-BEST-TROSY-HSQC spectra of PilF_159-221_ WT in complex with c-di-GMP on PilF_159-302_ WT in complex with c-di-GMP. (c) Chemical shift changes induced by c-di-GMP binding to PilF_159-221_ WT. Overlay of the ^15^N-BEST-TROSY-HSQC spectra of free and c-di-GMP-bound PilF_159-221_ WT. (d) Chemical shift changes induced by c-di-GMP binding to PilF_159-302_ WT. Overlay of the ^15^N-BEST-TROSY-HSQC spectra of free and c-di-GMP-bound PilF_159-302_ WT.


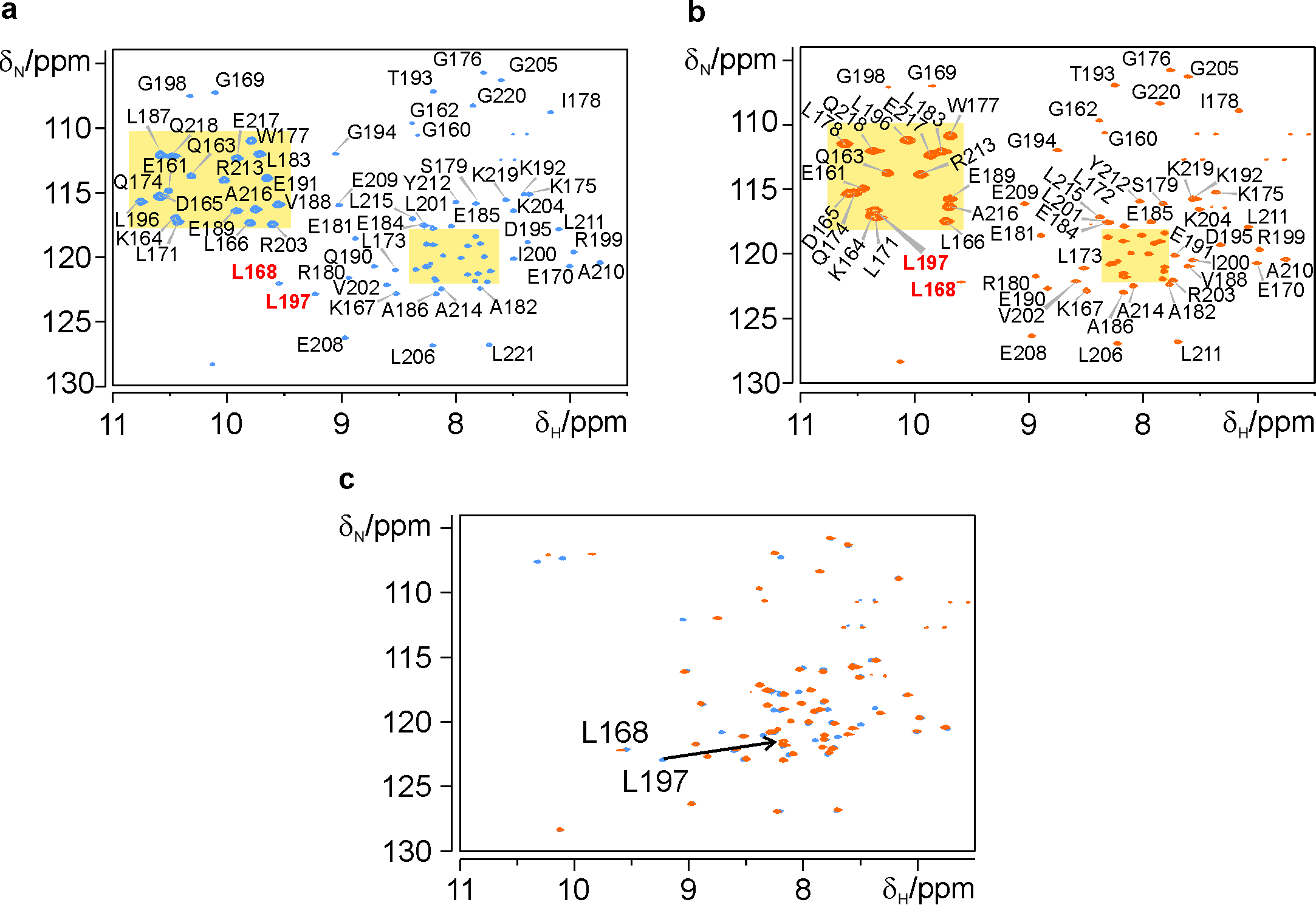


**Figure S6: Backbone assignments of PilF_159-221_ WT and PilF_159-221_ Q190E in complex with c-di-GMP.** (a, b) ^15^N-BEST-TROSY-HSQC spectrum of PilF_159-221_ WT (a) and PilF_159-221_ Q190E (b) in complex with c-di-GMP. Assignments are shown in one letter code with the respective residue numbers. L168 and L197 assignments are highlighted in red and bold letters. In both spectra crowded areas are magnified in separate areas highlighted by yellow backgrounds. (c) Overlay of the ^15^N-Best-TROSY-HSQC spectra of c-di-GMP-bound PilF_159-221_ WT (blue) and Q190E (orange) variant. A black arrow indicates the chemical shift change for the amide group signal of L197.


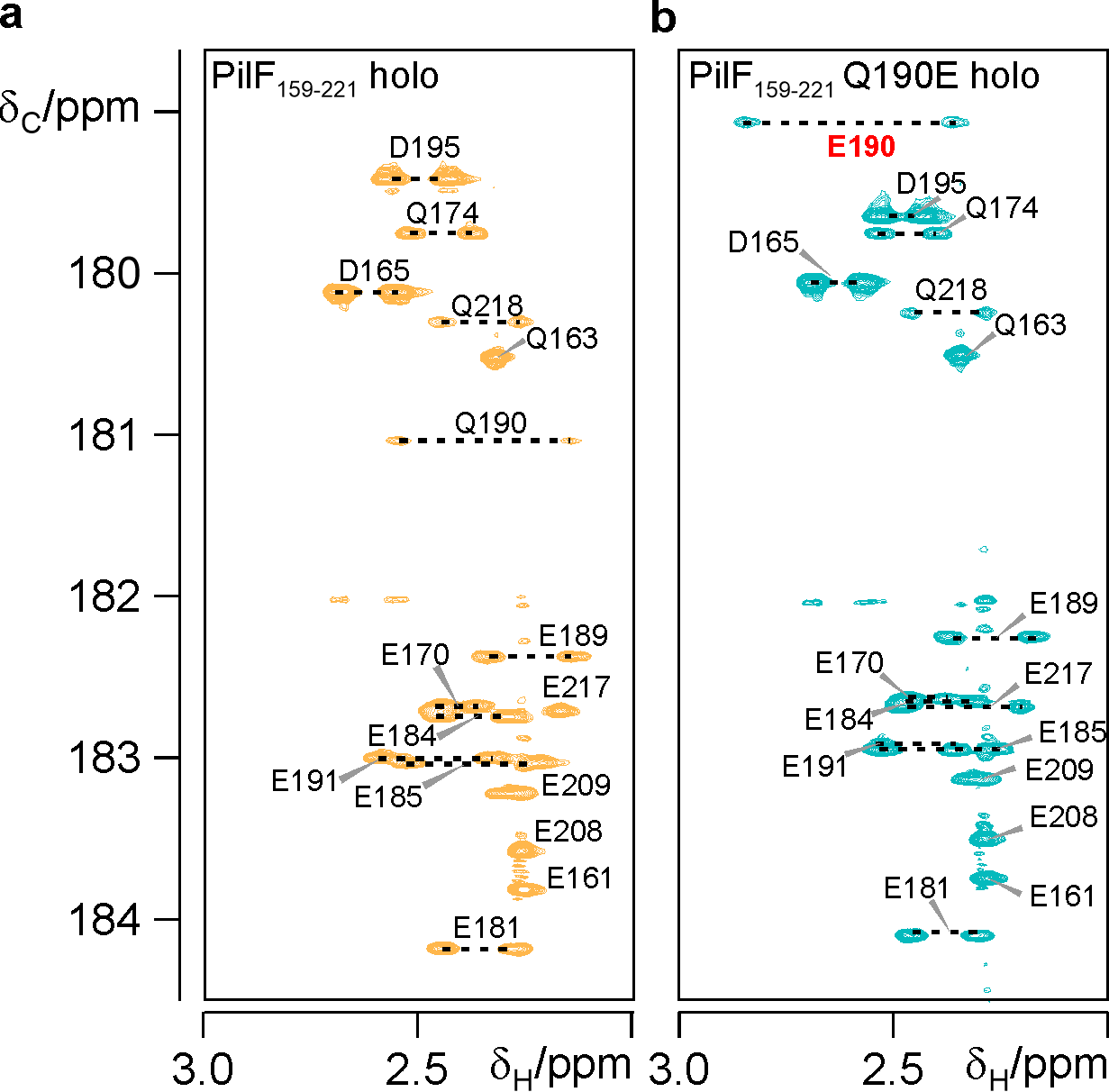


**Figure S7: Chemical shift correlations of PilF_159-221_ WT and mutant glutamate/glutamine Hγ protons with Cδ carbon atoms and aspartate Hβ protons with the Cγ carbons.** (a) 2D-Hγ(Cγ)Cδ spectrum of PilF_159-221_ WT in complex with c-di-GMP. (b) 2D-Hγ(Cγ)Cδ spectrum of PilF_159-221_ Q190E in complex with c-di-GMP. Black dashed lines indicate resonance pairs belonging to protons from the same side chain methylene group.


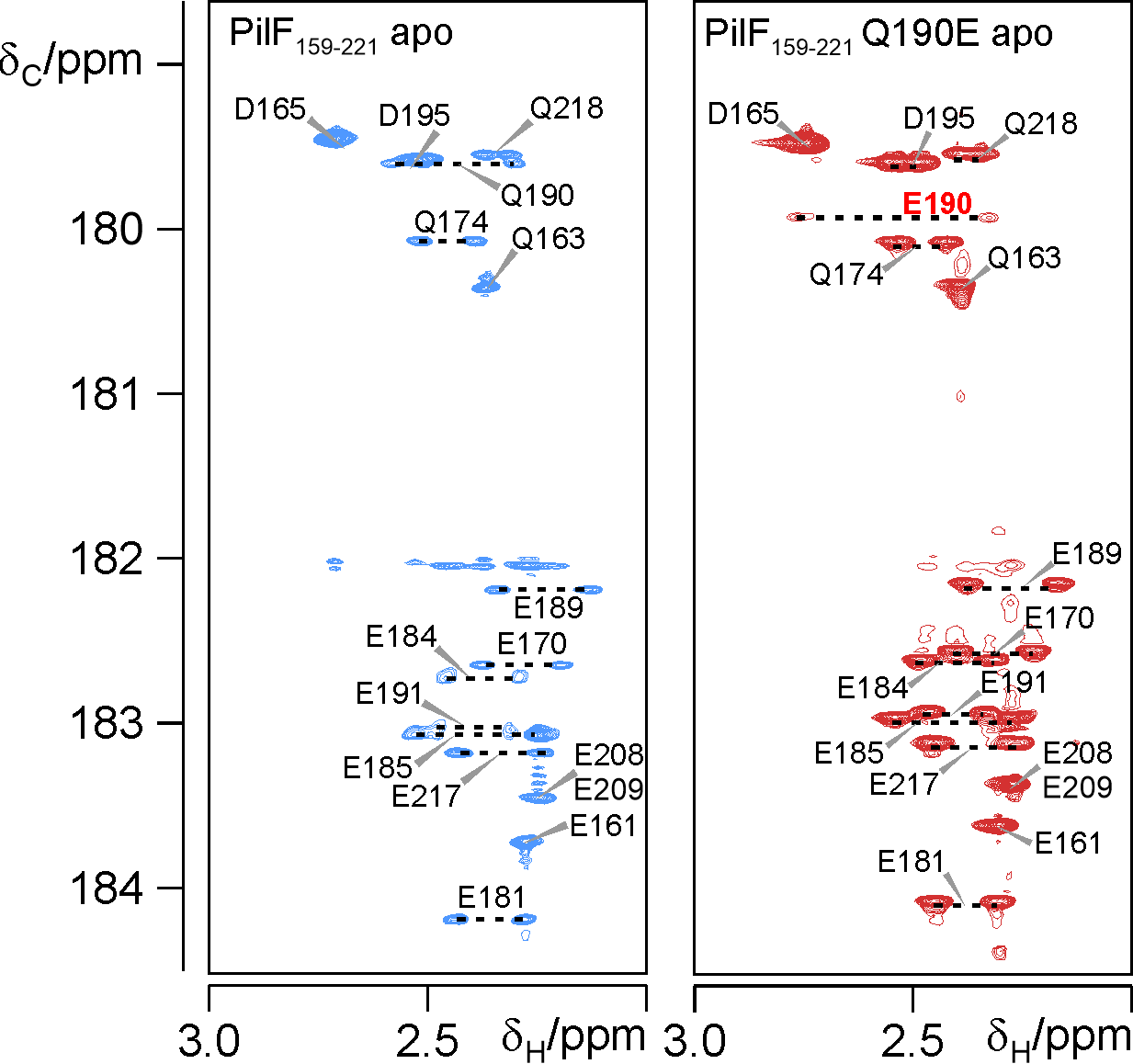


**Figure S8: Correlations of the chemical shifts of the Hγ protons with the Cδ carbon atoms of glutamate/glutamine and the Hβ protons with the Cγ carbons of aspartate for free PilF_159-221_ WT and free PilF_159-221_ Q190E.** (a) 2D-Hγ(Cγ)Cδ NMR spectra of free PilF_159-221_ WT. (b) 2D-Hγ(Cγ)Cδ NMR spectra of free PilF_159-221_ Q190E. Black dashed lines indicate resonance pairs belonging to protons from the same side chain methylene group.


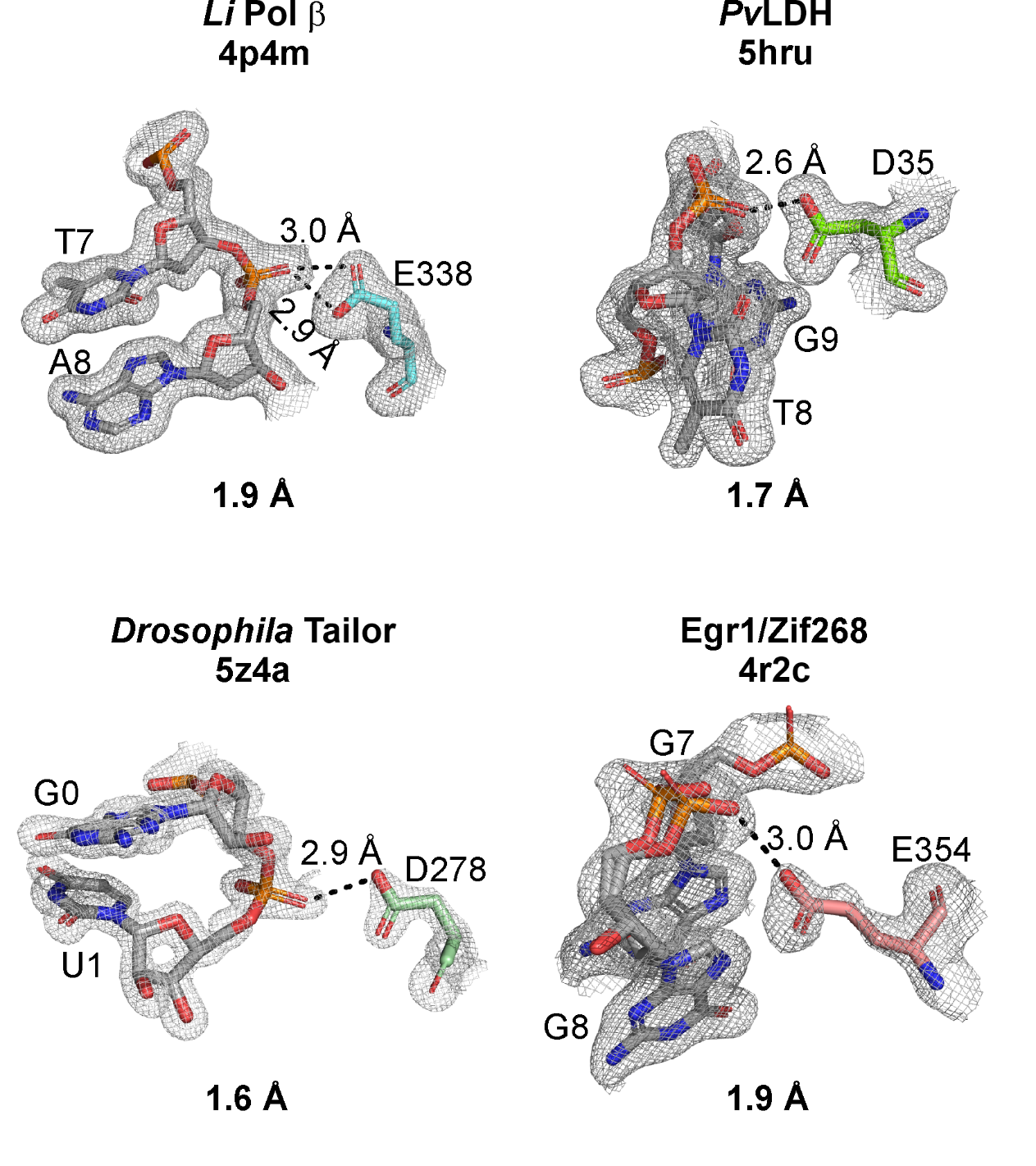


**Figure S9: Additional examples of glutamate/aspartate carboxylate groups in phosphodiester group recognition by DNA/RNA binding proteins.** Shown are examples from crystal structures for a DNA polymerase (PDB: 4P4M) ^[37]^, a lactate dehydrogenase (PDB: 5HRU) ^[38]^, a terminal uridylyl transferase (PDB: 5Z4A) ^[39]^, and a transcription factor (PDB: 4R2C) ^[40]^ in complex with a DNA or an RNA ligand. For each example the protein name, the respective pdb code and the obtained resolution is given. Hydrogen bonds are indicated as black dashed lines and electron density is shown as a grey mesh at a σ level of 1.0 except for Drosophila Tailor, which has a mesh at a σ level of 2.0.

**Material & Methods**

**Plasmids and construct design**

For protein production and construct design, a commercially obtained pET-11a plasmid (GenScript, New Jersey, USA) encoding for PilF_159-302_ with an N-terminal hexahistidine (His-6) tag and a TEV-cleavage site was used. Point mutations (Q190E and Q218E) were introduced using a standard two-step side directed mutagenesis protocol ^[41]^. C-terminally truncated constructs (PilF_159-221_) were created by standard Gibson assembly ^[42]^.

**Protein and ligand preparation**

All protein constructs were heterologously expressed in the *E. coli* BL21 [DE3] *Gold* cell line (Agilent Technologies/Stratagen) with an N-terminal His-6 tag followed by a TEV-cleavage site. TEV-cleavage resulted in 144 (PilF_159-302_) or 63 (PilF_159-221_) native residues and two artificial residues (G and S) from the TEV-cleavage site including the point-mutation variants (Q190E and Q218E). Protein production and purification of unlabeled and uniformly ^15^N or ^13^C,^15^N labelled protein constructs was performed as described before ^[43]^.

To characterize c-di-GMP binding by PilF_159-302_, the Q190E and Q218E constructs via isothermal titration calorimetry (ITC) and X-ray crystallography unlabeled c-di-GMP was synthesized using a constitutively active mutant of a diguanylate cyclase from the thermophilic bacterium *Thermogata maritima* ^[44,45]^*.* For NMR experiments ^13^C,^15^N labelled c-di-GMP was synthesized identically using ^13^C,^15^N labelled GTP as a substrate.

**Crystallization**

Crystallization of PilF_159-302_ Q190E and PilF_159-302_ Q218E complexed with c-di-GMP was carried out using the hanging-drop-vapor-diffusion protocol in 24-well crystallization plates at 291 K. For PilF_159-302_ Q190E 1.6 M LiSO_4_, 0.1 M sodium acetate pH 7.0 and for PilF_159-302_ Q218E 1.5 M LiSO_4_, 0.1 M sodium acetate pH 7.0 was used as a precipitant with a volume of 500 µl. During harvesting, the crystals were flash-frozen in liquid nitrogen without any further cryo-protection.

**Diffraction data collection and refinement**

All crystals in this study were tested for diffraction at the P13 beamline of Deutsches Elektronen-Synchrotron (DESY), Hamburg, Germany. Data was collected at 100 K with a wavelength of 0.976 Å and processed with the XDS processing software ^[46]^ for indexing and integration. All datasets were merged with Pointless and Aimless ^[47,48]^ implemented in the XDS processing software package resulting in overall completeness of 100% for both constructs and R_merge_ values of 10% for Q190E and 8% for Q218E. Molecular replacement was performed with the X-ray structure of PilF_159-302_ in complex with c-di-GMP (PDB 8PDK) as a poly-alanine model without c-di-GMP in PHENIX ^[49]^. Subsequent iterative model rebuilding, refinement and quality assessment was performed using PHENIX and WinCoot ^[50,51]^.

**NMR spectroscopy**

All NMR-samples were prepared in 50 mM Bis-Tris pH 5.8, 200 mM NaCl, 1 mM β-Mercaptoethanol (β-Me) and 5% (v/v) D_2_O. To reference proton resonances each sample contained 200 µM 2,2-dimethyl-2-silapentane-5-sulfuric acid (DSS). ^15^N and ^13^C chemical shifts were indirectly referenced to the DSS resonance using the appropriate conversion factors ^[52]^. NMR-spectra were recorded on Bruker AVANCE 600, 700, 800, 900, and 950 MHz spectrometers equipped with cryogenic triple resonance probes and processed using TopSpin version 4.0.7. Chemical shifts were assigned in CARA (Keller, R. L. J.) For the backbone and sidechain assignment of PilF_159-221_ and the mutant PilF_159-221_ Q190E samples were prepared with concentrations of 700 µM protein for the apo states and 700 µM protein with 840 µM c-di-GMP (1.2x) for the holo states. A set of 3D-BEST-TROSY-HNCO and -HNCACB spectra for the backbone assignment and 3D-H(CCO)NH and -(H)C(CCO)NH spectra for the standard side chain assignment were recorded in H_2_O at 318 K.

To investigate carbon resonances of carboxylate groups (Asp, Glu) and carboxamide groups (Asn, Gln) via the Cβ/Cγ-CO correlation, samples were prepared with 700 µM ^13^C,^15^N labelled protein and 840 µM c-di-GMP. Two-dimensional H(CA)CO spectra ^[53,54]^ optimized for the detection of the Hγ(Cγ)Cδ and Hβ(Cβ)Cγ correlation ^[55,56]^ were recorded at 318 K at 600 MHz.

To assess hydrogen bond strength via cross-hydrogen bond scalar couplings between amide protons and phosphate groups (^2h^J_HP_) a set of 2D ^1^H{^31^P) spin-echo-^15^N-sofast-HMQC experiments with (cross) and without (reference) ^31^P decoupling ^[30,31]^ were recorded for PilF_159-221_ and the mutant PilF_159-221_ Q190E. To ensure comparability the same buffer conditions (50 mM BisTris pH 5.8, 200 mM NaCl, 1 mM β-Me) and temperature (298 K) were used at the same spectrometer (700 MHz). Four different spin-echo delays (25, 50, 75, 100 ms) were used for the cross and reference experiments and each experiment was recorded as a duplicate. Resonance integral ratios of the coupled (I_cross_) and the decoupled reference (I_ref_) were plotted against the constant-time delay (τ_m_). To calculate ^2h^J_HP_ values the data was fitted to Equation 1 (Eq. 1)

$$\frac{I_{cross}}{I_{ref}}=cos(\pi^{2h}J_{H,P}\tau_{m})$$

To investigate 2’-OH proton chemical shifts of c-di-GMP bound to PilF_159-221_ and the mutant PilF_159-221_ Q190E 2D-^13^C-HSQC-TOCSY spectra were recorded at 288 K with samples containing 700 µM unlabeled PilF_159-221_ or PilF_159-221_ Q190E and 840 µM uniformly ^13^C,^15^N-labelled c-di-GMP.

**Isothermal Titration Calorimetry (ITC)**

c-di-GMP binding capabilities of PilF_159-302,_ its mutant constructs PilF_159-302_ Q190E and PilF_159-302_ Q218E at varying pH-values were investigated by isothermal titration calorimetry (ITC) experiments in triplicate. All measurements were conducted at 20 °C using a MicroCal ITC_200_ (Malvern Panalytical, UK). For PilF_159-302_ Q190E and PilF_159-302_ Q218E ITC-measurements were conducted in a pH range from pH 8.5 – 4.0 with the buffering agent adjusted to the respective pH-range. For the pH-range 8.5 – 7.5 a sample buffer of 50 mM Tris, 200 mM NaCl and 1 mM β-Me was used. For pH values 7.0 – 6.0 a sample buffer of 50 mM BisTris, 200 mM NaCl, 1 mM β-Me and for pH 5.5 – 4.0 a sample buffer of 50 mM NaAc, 200 mM NaCl, 1 mM β-Me was used. For reference PilF_159-302_ was titrated with c-di-GMP at pH 8.5 in 50 mM Tris pH 8.5, 200 mM NaCl, 1 mM β-Me and at pH 4.0 in 50 mM NaAc pH 4.0, 200 mM NaCl, 1 mM β-Me. In order to test whether the buffer reagents have an influence on the measured thermodynamic parameters, cross measurements were performed in the region of the pH transitions. To this end, additional measurements with BisTris at pH 7.5 and 5.5, with Tris at pH 7.0 and with NaAc at pH 6.0 were carried out as singlets. The buffer agents had no influence on measured K_D_-values and resulted only in small changes of the ΔH values. All protein samples were dialyzed two times against 500 ml of the respective sample buffer (1 L in total) at 4 °C for at least 16 hours per step using a dialysis chamber with a nominal 3.5 kDa molecular weight cutoff (ZelluTrans, ROTH). To prepare the ITC-measurements protein and c-di-GMP sample were adjusted to the respective concentration using the dialysis buffer. Protein and c-di-GMP concentration ranged from 20 to 50 µM (protein) and 200 to 500 µM (c-di-GMP) depending on the c-di-GMP binding capabilities at the different pH-values to optimize precision of the measurements. For pH 8.5 – 7.0 a protein concentration of 50 µM with 500 µM c-di-GMP was used. For pH 6.5 – 5.0 a protein concentration of 30 µM with 300 µM c-di-GMP and for pH 4.5 – 4.0 a protein concentration of 20 µM with 200 µM c-di-GMP was used. The following protocol was utilized for each measurement. To 270 µl protein a total of 40 µl c-di-GMP was titrated in 21 injections with a duration of 4 s in intervals of 180 s with a stirring speed of 750 rpm. The first injection of 0.2 µl c-di-GMP with a duration of 0.4 s was carried out after an initial delay of 120 s, which was followed by 20 injections of 2 µl each with a duration of 4 s. During the measurement, the feedback mode was set to high and the reference power to 11 µcal s^-1^. All thermograms were processed using Origin 7.0 (OriginLab) using the one-site binding model.

To investigate pK_a_ values of the mutants PilF_159-302_ Q190E and PilF_159-302_ Q218E in the absence (pK_af_) and presence (pK_ab_) of c-di-GMP, the measured K_D_ values of the constructs in the pH-range of 8.5 to 4.0 were plotted against the pH-values. To calculate the pK_a_ values the data was fitted to Equation 2 (Eq. 2)

$$K_{D}=K_{b}\frac{1+{10}^{{({pK}_{a})}_{b}-pH}}{1+{10}^{{({pK}_{a})}_{f}-pH}}$$

using Origin 2019 (OriginLab).

**Bioinformatics**

A self-written Python script implementing the BioPandas module ^[57]^ was used to scan a local, time-stamped (most recent version in July 2024) copy of the PDB (protein protein data bank) hosted on NMRbox ^[58]^ under the ReBoxitory data source ^[59]^ for the potential interaction of protonated glutamate or aspartate side chains and phosphodiester groups.

A pre-selection was made by considering only entries containing X-ray structures of RNA-protein or DNA-protein complexes with a resolution better than 2.8 Å. The pre-selected atomic coordinate files were used to identify PDB entries with distances between the Oδ oxygens of glutamates, Oγ oxygens of aspartates and the OP1 and OP2 non-bridging oxygen atoms of phosphodiester groups, respectively, smaller than 3 Å. The initial list was further curated upon visual inspection to exclude entries of RNA/DNA-polymerases, nucleases, gyrases, ligases and other enzymes acting on nucleic acids which often contain aspartate and glutamate residues complexed to divalent metal ions in their active centers which are naturally in close proximity to the phosphodiester backbone due to their catalytic activities.

**References**

[1] P. Thaplyal, P. C. Bevilacqua, *Methods Enzymol* **2014**, 549, 189–219.

[2] K. Nadassy, S. J. Wodak, J. Janin, *Biochemistry* **1999**, 38, 1999–2017.

[3] S. A. Coulocheri, D. G. Pigis, K. A. Papavassiliou, A. G. Papavassiliou, *Biochimie* **2007**, 89, 1291–1303.

[4] S. Jones, P. van Heyningen, H. M. Berman, J. M. Thornton, *J Mol Biol* **1999**, 287, 877–896.

[5] R. P. Bahadur, M. Zacharias, J. Janin, *Nucleic Acids Res* **2008**, 36, 2705–2716.

[6] M. Treger, E. Westhof, *J Mol Recog* **2001**, 14, 199–214.

[7] S. Jones, D. T. A. Daley, N. M. Luscombe, H. M. Berman, J. M. Thornton, *Nucleic Acids Res* **2001**, 29, 943–954.

[8] X. Zeng, M. Huang, Q.-X. Sun, Y.-J. Peng, X. Xu, Y.-B. Tang, J.-Y. Zhang, Y. Yang, C.-C. Zhang, *Proc Natl Acad Sci USA* **2023**, 120, e2221874120.

[9] N. M. Luscombe, S. E. Austin, H. M. Berman, J. M. Thornton, *Genome Biol* **2000**, 1, reviews001.1-reviews001.37.

[10] D. E. Draper, *Annu Rev Biochem* **1995**, 64, 593–620.

[11] D. E. Draper, *J Mol Biol* **1999**, 293, 255–270.

[12] Y. Nozaki, C. Tanford, *J Biol Chem* **1967**, 242, 4731–4735.

[13] M. Tollinger, J. D. Forman-Kay, L. E. Kay, *J Am Chem Soc* **2002**, 124, 5714–5717.

[14] P. Keim, R. A. Vigna, J. S. Morrow, R. C. Marshall, F. R. N. Gurd, *J Biol Chem* **1973**, 248, 7811–7818.

[15] R. Simm, M. Morr, A. Kader, M. Nimtz, U. Römling, *Mol Microbiol* **2004**, 53, 1123–1134.

[16] A. J. Wolfe, K. L. Visick, J Bacteriol 2008, 190, 463–475.

[17] C. R. Guzzo, R. K. Salinas, M. O. Andrade, C. S. Farah, *J Mol Biol* **2009**, 393, 848–866.

[18] R. Tamayo, J. T. Pratt, A. Camilli, *Annu Rev Microbiol* 2007, **61**, 131–148.

[19] R. Hengge, *Nat Rev Microbiol* **2009**, 7, 263–273.

[20] R. Salzer, F. Joos, B. Averhoff, *Appl Environ Microbiol* **2014**, 80, 644–652.

[21] K. Kruse, R. Salzer, F. Joos, B. Averhoff, *Extremophiles* **2018**, 22, 461–471.

[22] K. Neißner, H. Keller, L. Kirchner, S. Düsterhus, E. Duchardt-Ferner, B. Averhoff, J. Wöhnert, *J Biol Chem* **2025**, 301, 108041.

[23] Y.-C. Wang, K.-H. Chin, Z.-L. Tu, J. He, C. J. Jones, D. Z. Sanchez, F. H. Yildiz, M. Y. Galperin, S.-H. Chou, *Nat Commun* **2016**, 7, 12481.

[24] J. C.-H. Chen, B. L. Hanson, S. Z. Fisher, P. Langan, A. Y. Kovalevsky, *Proc Natl Acad Sci USA* **2012**, 109, 15301–15306.

[25] M. A. S. Hass, F. A. A. Mulder, *Ann Rev Biophys* **2015**, 44, 53–75.

[26] M. Betz, F. Löhr, H. Wienk, H. Rüterjans, *Biochemistry* **2004**, 43, 5820–5831.

[27] M. Pellecchia, H. Iwai, T. Szyperski, K. Wüthrich, *J Magn Reson* **1997**, 124, 274–278.

[28] J. M. Bradshaw, G. Waksman, *Biochemistry* **1998**, 37, 15400–15407.

[29] W. R. Forsyth, J. M. Antosiewicz, A. D. Robertson, *Proteins* **2002**, 48, 388–403.

[30] E. Duchardt-Ferner, J. Ferner, J. Wöhnert, *Angew Chem Int Ed Engl* **2011**, 50, 7927–7930.

[31] E. Duchardt-Ferner, J. Wöhnert, *J Biomol NMR* **2017**, 69, 101–110.

[32] M. Hennig, J. Fohrer, T. Carlomagno, *J Am Chem Soc* **2005**, 127, 2028–2029.

[33] J.-P. Surivet, R. Lange, C. Hubschwerlen, W. Keck, J.-L. Specklin, D. Ritz, D. Bur, H. Locher, P. Seiler, D. S. Strasser, L. Prade, C. Kohl, C. Schmitt, G. Chapoux, E. Ilhan, N. Ekambaram, A. Athanasiou, A. Knezevic, D. Sabato, A. Chambovey, M. Gaertner, M. Enderlin, M. Boehme, V. Sippel, P. Wyss, *Bioorg Med Chem Lett* **2012**, 22, 6705–6711.

[34] M. Sokolowska, M. Kaus-Drobek, H. Czapinska, G. Tamulaitis, R. H. Szczepanowski, C. Urbanke, V. Siksnys, M. Bochtler, *J Mol Biol* **2007**, 369, 722–734.

[35] D. G. Sashital, M. Jinek, J. A. Doudna, *Nat Struct Mol Biol* **2011**, 18, 680–687.

[36] N. Czudnochowski, G. W. Ashley, D. V. Santi, A. Alian, J. Finer-Moore, R. M. Stroud, *Nucleic Acids Res* **2014**, 42, 2037–2048.

[37] E. Mejia, M. Burak, A. Alonso, V. Larraga, T. A. Kunkel, K. Bebenek, M. Garcia-Diaz *DNA Repair* **2014**, 18, 1-9

[38] S-J. Choi, C. Ban *Sci Rep* **2016**, 11, 34998

[39] L. Cheng, F. Li, Y. Jiang, H. Yu, C. Xie, Y. Shi, Q. Gong *Nucleic Acids Res* **2018**, 47, 495-508

[40] H. Hashimoto, Y. O. Olanrewaju, Y. Zheng, G. G. Wilson, X. Zhang, X. Cheng *Genes Dev* **2014**, 28, 2304-2313

[41] O. Edelheit, A. Hanukoglu, I. Hanukoglu, *BMC Biotechnol* **2009**, 9, 61.

[42] D. G. Gibson, L. Young, R.-Y. Chuang, J. C. Venter, C. A. Hutchison, H. O. Smith, *Nat Methods* **2009**, 6, 343–345.

[43] K. Neißner, H. Keller, E. Duchardt-Ferner, C. Hacker, K. Kruse, B. Averhoff, J. Wöhnert, *Biomol NMR Assign* **2019**, 13, 383–390.

[44] F. Rao, S. Pasunooti, Y. Ng, W. Zhuo, L. Lim, A. W. Liu, Z.-X. Liang, *Anal Biochem* **2009**, 389, 138–142.

[45] H. Keller, A. K. Weickhmann, T. Bock, J. Wöhnert, *RNA* **2018**, 24, 1390–1402.

[46] W. Kabsch, *Acta Cryst D* **2010**, 66, 133–144.

[47] P. Evans, *Acta Cryst D* **2006**, 62, 72–82.

[48] P. R. Evans, *Acta Cryst D* **2011**, 67, 282–292.

[49] D. Liebschner, P. V. Afonine, M. L. Baker, G. Bunkóczi, V. B. Chen, T. I. Croll, B. Hintze, L.-W. Hung, S. Jain, A. J. McCoy, N. W. Moriarty, R. D. Oeffner, B. K. Poon, M. G. Prisant, R. J. Read, J. S. Richardson, D. C. Richardson, M. D. Sammito, O. V. Sobolev, D. H. Stockwell, T. C. Terwilliger, A. G. Urzhumtsev, L. L. Videau, C. J. Williams, P. D. Adams, *Acta Cryst D* **2019**, 75, 861–877.

[50] P. V. Afonine, R. W. Grosse-Kunstleve, N. Echols, J. J. Headd, N. W. Moriarty, M. Mustyakimov, T. C. Terwilliger, A. Urzhumtsev, P. H. Zwart, P. D. Adams, *Acta Cryst D* **2012**, 68, 352–367.

[51] C. J. Williams, J. J. Headd, N. W. Moriarty, M. G. Prisant, L. L. Videau, L. N. Deis, V. Verma, A. Keedy, B. J. Hintze, V. B. Chen, S. Jain, S. M. Lewis, W. B. Arendall, J. Snoeyink, P. D. Adams, S. C. Lovell, J. S. Richardson, D. C. Richardson, *Protein Sci* **2018**, 27, 293–315.

[52] J. L. Markley, A. Bax, Y. Arata, C. W. Hilbers, R. Kaptein, B. D. Sykes, P. E. Wright, K. Wüthrich, *J Mol Biol* **1998**, 70, 117–142.

[53] R. Powers, A. M. Gronenborn, G. Marius Clore, A. Bax, *J Magn Reson* **1991**, 94, 209–213.

[54] S. Grzesiek, A. Bax, *J Magn Reson Series B* **1993**, 102, 103–106.

[55] M. Betz, F. Löhr, H. Wienk, H. Rüterjans, *Biochemistry* **2004**, 43, 5820–5831.

[56] M. Pellecchia, H. Iwai, T. Szyperski, K. Wüthrich, *J Magn Reson* **1997**, 124, 274–278.

[57] S. Raschka, *JOSS* **2017**, 2, 279.

[58] M. W. Maciejewski, A. D. Schuyler, M. R. Gryk, I. I. Moraru, P. R. Romero, E. L. Ulrich, H. R. Eghbalnia, M. Livny, F. Delaglio, J. C. Hoch, *Biophys J* **2017**, 112, 1529–1534.

[59] K. Baskaran, D. L. Craft, H. R. Eghbalnia, M. R. Gryk, J. C. Hoch, M. W. Maciejewski, A. D. Schuyler, J. R. Wedell, C. W. Wilburn, *Front Mol Biosci* **2022**, 8.
